# Atherosclerosis amelioration by allicin in raw garlic through gut microbiota and trimethylamine-*N*-oxide modulation

**DOI:** 10.1038/s41522-022-00266-3

**Published:** 2022-01-27

**Authors:** Suraphan Panyod, Wei-Kai Wu, Pei-Chen Chen, Kent-Vui Chong, Yu-Tang Yang, Hsiao-Li Chuang, Chieh-Chang Chen, Rou-An Chen, Po-Yu Liu, Ching-Hu Chung, Huai-Syuan Huang, Angela Yu-Chen Lin, Ting-Chin David Shen, Kai-Chien Yang, Tur-Fu Huang, Cheng-Chih Hsu, Chi-Tang Ho, Hsien-Li Kao, Alexander N. Orekhov, Ming-Shiang Wu, Lee-Yan Sheen

**Affiliations:** 1 Institute of Food Science and Technology, National Taiwan University, Taipei, Taiwan; 2 Department of Medical Research, National Taiwan University Hospital, Taipei, Taiwan; 3 Department of Internal Medicine, National Taiwan University Hospital, Taipei, Taiwan; 4 National Laboratory Animal Center, National Applied Research Laboratories, Taipei, Taiwan; 5 Department of Internal Medicine, College of Medicine, National Taiwan University, Taipei, Taiwan; 6 Department of Medicine, Mackay Medical College, New Taipei City, Taiwan; 7 Graduate Institute of Environmental Engineering, National Taiwan University, Taipei, Taiwan; 8 Division of Gastroenterology, Perelman School of Medicine, University of Pennsylvania, Philadelphia, PA USA; 9 Department and Graduate Institute of Pharmacology, National Taiwan University College of Medicine, Taipei, Taiwan; 10 Research Center for Developmental Biology & Regenerative Medicine, National Taiwan University, Taipei, Taiwan; 11 Division of Cardiology, Department of Internal Medicine and Cardiovascular Center, National Taiwan University Hospital, Taipei, Taiwan; 12 Institute of Biomedical Sciences, Academia Sinica, Taipei, Taiwan; 13 Department of Chemistry, National Taiwan University, Taipei, Taiwan; 14 Department of Food Science, Rutgers University, New Brunswick, NJ USA; 15 Institute for Atherosclerosis Research, Skolkovo Innovative Center, Moscow, Russia; 16 Laboratory of Angiopathology, Institute of General Pathology and Pathophysiology, Moscow, Russia; 17 Center for Food and Biomolecules, National Taiwan University, Taipei, Taiwan; 18 National Center for Food Safety Education and Research, National Taiwan University, Taipei, Taiwan

**Keywords:** Microbiome, Health care

## Abstract

Cardiovascular disease (CVD) is strongly associated with the gut microbiota and its metabolites, including trimethylamine-*N*-oxide (TMAO), formed from metaorganismal metabolism of ʟ-carnitine. Raw garlic juice, with allicin as its primary compound, exhibits considerable effects on the gut microbiota. This study validated the benefits of raw garlic juice against CVD risk via modulation of the gut microbiota and its metabolites. Allicin supplementation significantly decreased serum TMAO in ʟ-carnitine-fed C57BL/6 J mice, reduced aortic lesions, and altered the fecal microbiota in carnitine-induced, atherosclerosis-prone, apolipoprotein E-deficient (ApoE^−/−^) mice. In human subjects exhibiting high-TMAO production, raw garlic juice intake for a week reduced TMAO formation, improved gut microbial diversity, and increased the relative abundances of beneficial bacteria. In in vitro and ex vivo studies, raw garlic juice and allicin inhibited γ-butyrobetaine (γBB) and trimethylamine production by the gut microbiota. Thus, raw garlic juice and allicin can potentially prevent cardiovascular disease by decreasing TMAO production via gut microbiota modulation.

## Introduction

Cardiovascular disease (CVD) is the primary cause of global mortality and is responsible for approximately one-third of all deaths worldwide^1,2^. Consumption of an unhealthy diet, excessive alcohol use, smoking, and lack of physical activity are considered traditional health risk factors for CVD^3^. Recently, an insidious CVD risk factor has been explored; the gut microbiota is now considered an endocrine organ that communicates with the body through the gut-systemic axis and is extensively modifiable by foods^4–6^. The gut microbiota utilizes nutrients from undigested foods for growth and produces miscellaneous metabolites that regulate host–microbe homeostasis, which may be involved in the pathogenesis of cardiometabolic diseases^7–9^. For example, phosphatidylcholine, choline, and ʟ-carnitine from egg, dairy products, and red meat can be metabolized by specific gut bacteria to produce trimethylamine (TMA), which is subsequently oxidized to trimethylamine-N-oxide (TMAO) by hepatic flavin monooxygenase^10–12^. Increased concentrations of blood TMAO have a strong link with increased major adverse cardiovascular events and all-cause mortality^13,14^. The mechanisms underlying the atherogenic and thrombogenic effects of TMAO include enhanced foam cell formation, reduced reverse cholesterol transport, and induction of platelet aggregation^9,10,12,15^. TMAO has been reported to enhance platelet aggregation and induce thrombosis in both in vitro and human studies^16,17^. The following multistep microbial pathway was recently proposed as the principal route for TMAO production through carnitine metabolism by the gut microbiota: ʟ-carnitine → γ-butyrobetaine (γBB) → TMA → TMAO^18,19^. A gut bacterium, *Emergencia timonensis*, was found to be capable of transforming γBB to TMA anaerobically and was reported to partially explain the phenotype of high-TMAO producers among human beings^18,20^.

Investigations on how different food components interact with the gut microbiota have improved our understanding of modifying our dietary behavior to achieve a health-promoting state^8,21^. Certain approaches were proposed for reducing TMAO production by the gut microbiota such as antibiotic use, fecal microbiota transplantation, and administration of TMA lyase inhibitor^7,9,22–24^. However, these approaches might exhibit safety concerns and require validation in human studies. Several compounds found in foods and herbs exhibit anti-atherosclerosis activity via the inhibition of commensal microbial TMA production^25–28^. Garlic (*Allium sativum* L.) has a long history of use as a spice in human food^29^. Traditionally, the purpose of adding garlic to meat in processed foods is not only to improve flavor but also to extend shelf life. In herbal medicine, garlic has been used as a dietary therapy against cardiovascular and other metabolic diseases^30–33^. It has been widely used as a natural antibiotic on the basis of its broad-spectrum antimicrobial property^34^. Allicin is the primary compound in raw garlic puree; it is produced through the conversion of alliin by alliinase when the clove is crushed^35^. Allicin exhibits various antimicrobial activities in both in vitro and in vivo studies^35–37^. Recently, allicin was reported to exhibit a modulatory effect on the gut microbiota and reduced hepatic steatosis^38,39^. Additionally, our previous pilot study showed that allicin supplementation shaped the gut microbiota composition and reduced TMAO production by the gut microbiota in mice subject to moderate carnitine consumption (0.02% ʟ-carnitine)^40^. However, the role of raw garlic with allicin in improving cardiovascular phenotypes via gut microbiota modulation has not been fully elucidated. Therefore, in this study, we aimed to investigate the effect of allicin and raw garlic in modulating both the function and composition of the gut microbiota for cardiovascular protection. We investigated the TMAO-reducing and gut microbiota-modulating effects of allicin in raw garlic in both rodent models and humans with higher carnitine consumption. We additionally compared the anti-TMAO and anti-atherosclerotic efficacies of allicin with that of the TMA lyase inhibitor 3,3-dimethyl-1-butanol (DMB)^24^, which has been previously shown to reduce serum TMAO in carnitine-treated mice^17^, through studies in both wild-type and apolipoprotein E-deficient (ApoE^−/−^) mice. Finally, we examined the effect of allicin-containing raw garlic juice on the TMA-producing activity of the human gut microbiota and specific gut bacteria responsible for multistep TMAO production.

## Results

### Allicin significantly reduced TMA, TMAO, and γBB levels in mice, with less effect on the gut microbiota compared with ʟ-carnitine

**Fig. 1:**
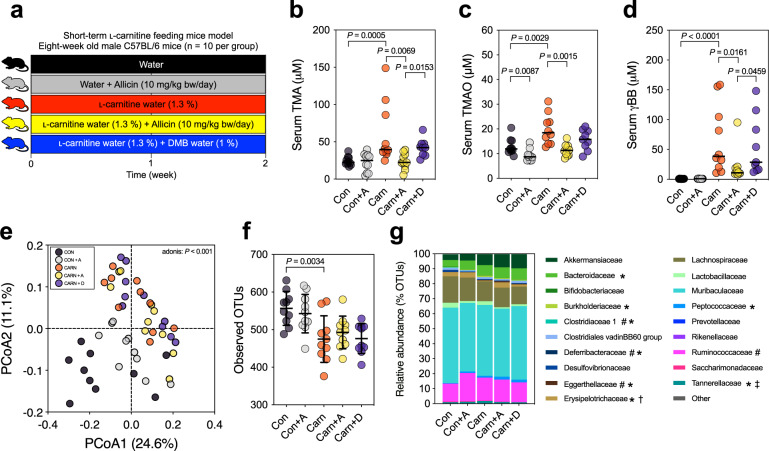
Allicin reduced serum TMA, TMAO, and γBB levels, and ʟ-carnitine principally changed the fecal microbiome composition in ʟ-carnitine-fed male C57BL/6 J mice (*n* = 10 per group). **a** Experimental design; **b** serum TMA; **c** serum TMAO; **d** serum γBB; **e** principal coordinate analysis (PCoA) plot with Bray−Curtis dissimilarity; **f** observed OTUs α-diversity indices; and **g** relative abundance of fecal microbiota at family level. Dot plots are expressed as the mean ± SD or median; statistical analyses of serum bacterial metabolites were performed using the Wilcoxon signed-rank test Con vs. Con + A group, Con vs. Carn group; Kruskal–Wallis tests with Dunn’s multiple comparisons, Carn vs. Carn + A group, Carn vs. Carn + D group, and Carn + A vs. Carn + D group. Gut microbiota ɑ-diversity was determined using the unpaired two-tailed Student’s *t*-test; one-way ANOVA with Tukey’s range test. The statistical analyses of the relative abundance were performed using the unpaired Wilcoxon signed-rank test with the false discovery rate (FDR), Con vs. Con + A group (^#^*P* < 0.1); Con vs. Carn group (*P < 0.1); Carn vs. Carn + A group (^†^*P* < 0.1); Carn + D group (^‡^*P* < 0.1).

To investigate the protective effect of allicin against CVD, C57BL/6 J mice were subject to increased daily consumption of ʟ-carnitine (1.3% in water) to examine the TMAO-reducing efficacy of allicin at a higher carnitine dosage. Additionally, we compared the TMAO-reducing effect of allicin with DMB (Fig. 1a). There was no significant change in the body weight of the mice (Supplementary Fig. 1). Serum TMA, TMAO, and γBB levels were increased in the carnitine-supplement group compared with the control (Fig. 1b–d). Serum TMA and TMAO levels were significantly reduced in the allicin-supplemented groups than those in the carnitine-supplement group (*P* = 0.0069 and *P* = 0.0015, respectively) (Fig. 1b, c). Additionally, serum γBB level was reduced in the allicin-supplemented group (*P* = 0.0161) (Fig. 1d). Allicin exhibited a better capability to reduce serum TMA and γBB levels than DMB. According to the microbiome analysis, a shift in the overall microbial composition was driven primarily by ʟ-carnitine treatment (adonis: *P* < 0.001) (Fig. 1e), whereas the gut microbiota composition did not vary considerably among the carnitine, carnitine + allicin, and carnitine + DMB groups. Additionally, the observed operational taxonomic units (OTUs) and Chao1 index were decreased in the ʟ-carnitine treatment groups (*P* = 0.0034; *P* = 0.0401, respectively), whereas supplementation with allicin and DMB did not reverse the ɑ-diversity (Fig. 1f and Supplementary Fig. 2b). The family-level relative abundance bar plot and OTU-level heatmap supported that ʟ-carnitine primarily shaped the gut microbial composition with respect to specific taxa (Fig. 1g and Supplementary Fig. 2c).

### Raw garlic juice reduced the TMAO-producing capacity and modified the gut microbiota by enriching beneficial gut bacteria in humans

**Fig. 2:**
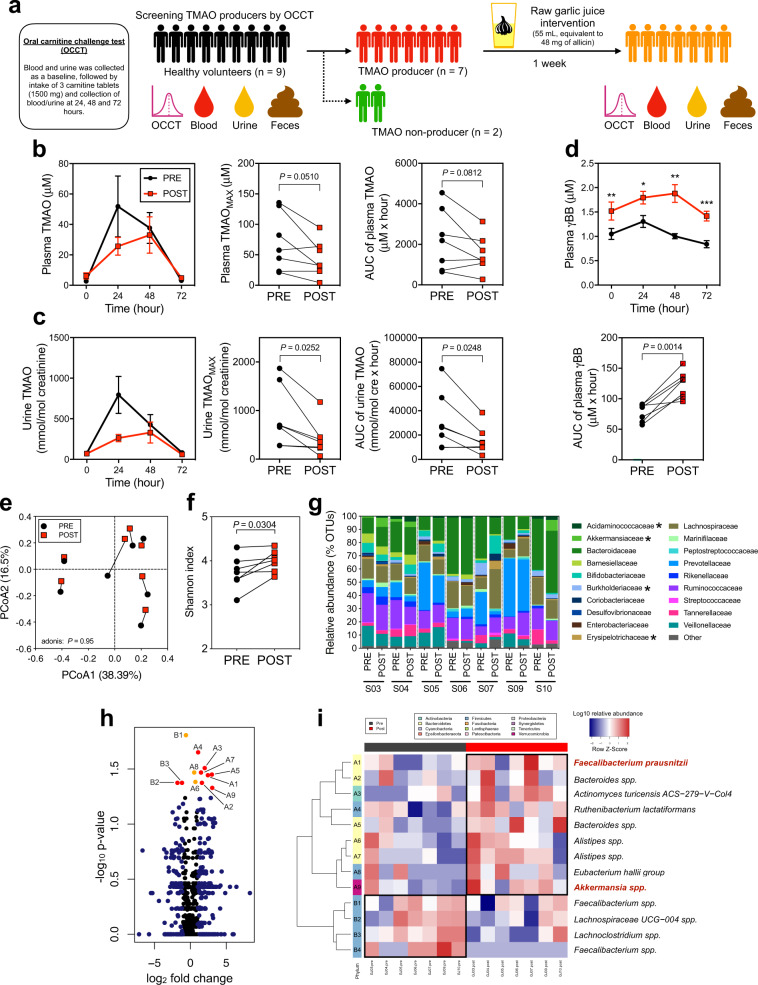
Raw garlic juice reduced plasma TMAO formation ability, increased plasma γBB level in healthy TMAO producers, and shaped fecal microbiota composition via increasing the evenness, ɑ-diversity, and relative abundance of specific beneficial bacterial taxa. **a** Experimental design, healthy participants (*n* = 9) were screened for TMAO production using oral carnitine challenge test (OCCT), the criterion for categorization as high-TMAO producers was plasma TMAO_MAX_ > 10 μM. High-TMAO producers (*n* = 7) subsequently received an intervention of garlic juice (55 mL, equivalent to 48 mg of allicin/day) for 1 week, followed by OCCT; **b** plasma and **c** urine TMAO, TMAO_MAX_, and TMAO_AUC_; **d** plasma γBB and γBB_AUC_. **e** Principal coordinate analysis (PCoA) plot with Bray−Curtis dissimilarity; **f** Shannon α-diversity index; **g** family-level composition of fecal microbiome; **h** volcano plot of fecal microbiota before and after garlic juice intervention; and **i** heatmap of the relative abundances of fecal microbiota with a significant difference using the Wilcoxon signed-rank test (*P* < 0.05). Data are expressed as the mean ± SEM; statistical analyses were performed by using the two-tailed paired Student’s *t*-test (*P < 0.05; **P < 0.01; and ***P < 0.001). The relative abundance bar plot statistical analysis was performed using a paired Wilcoxon signed-rank test (*P < 0.05). Volcano plot: red points indicate OTUs with a *P*-value < 0.05 and log_2_ fold-change > 1; orange points indicate OTUs with a *P*-value < 0.05; and blue points indicate log_2_ fold-change > 1.

On the basis of the TMAO-reducing effects of allicin observed in previous murine studies, we conducted a human pilot study to investigate whether raw garlic juice at an allicin-equivalent dosage could reduce the TMAO-producing capacity in the human body. We performed the oral carnitine challenge test (OCCT) which we established previously to evaluate TMAO-producing capacity in humans^41^. Nine volunteers were recruited to receive the OCCT, and garlic juice was administered to those who were high-TMAO producers (plasma TMAO_MAX_ ≥ 10 μM) according to the OCCT. Among the nine participants, seven were high-TMAO producers (Plasma TMAO_MAX_ = 130.91, 82.28, 57.53, 22.84, 135.62, 44.41, and 21.31 μM) (Fig. 2a and Supplementary Fig. 3a) and were selected for the garlic juice intervention study. We converted the allicin dose used in the murine study into a dose for humans and calculated the amount of blended garlic juice containing the equivalent dose of allicin for the interventional study. The blended raw garlic juice used in this pilot study contained 0.89 mg/mL allicin, and the participants consumed 55 mL of the prepared garlic juice per day for a week (Supplementary Fig. 4). The participants consumed raw garlic juice with a meal under supervision. We received feedback from all the participants that the intake of raw garlic juice with an empty stomach caused abdominal discomfort and stomachache. All the participants received the second OCCT after the 1-week raw garlic juice intervention. The area under the curve (AUC) and the maximum plasma/urine TMAO and γBB levels were calculated on the basis of the metabolites-time curve that reflected the gut microbiota-host response to carnitine oral intake and to observe changes after garlic juice administration. For the comparison of TMAO-producing capacity before and after the garlic juice intervention, a trend of reduced plasma TMAO_MAX_ and TMAO_AUC_ was observed (*P* = 0.0510; *P* = 0.0812) (Fig. 2b). Notably, both urine TMAO_MAX_ and TMAO_AUC_ were significantly reduced after the 1-week garlic juice intervention (*P* = 0.0252; *P* = 0.0248) (Fig. 2c). The strong correlation between plasma and urine TMAO concentrations (Supplementary Fig. 5a) supports the use of urine samples for measuring the TMAO-producing capacity. Interestingly, plasma levels of γBB in the OCCT were significantly increased (*P* < 0.01) (Fig. 2d), suggesting that the bioactive compounds in garlic may prevent the microbial transformation of γBB into TMA, thus decreasing TMAO formation. Our study showed a trend of reduction in platelet aggregation following the garlic juice intervention. Additionally, the maximum amplitude of platelet aggregation tended to exhibit a positive correlation with the TMAO levels (Supplementary Fig. 5b-c). Garlic-juice intervention did not lead to toxicity based on plasma biochemistry results (Supplementary Table 1). Only aspartate aminotransferase (AST) and alanine aminotransferase (ALT) were slightly increased but remained within the normal range.

Overall, the gut microbial composition was not altered by the 1-week consumption of garlic juice, as demonstrated through principal coordinate analysis (PCoA) (Fig. 2e). The intra-individual dissimilarity in the gut microbiome before and after treatment was considerably lower than the inter-individual index (*P* < 0.0001), suggesting that a relatively minor portion of the gut microbiota was altered by the garlic juice intervention (Supplementary Fig. 6a). Regarding the alpha diversity, garlic juice intervention significantly increased the Shannon index (*P* = 0.0304) (Fig. 2f). At the family level, the fecal microbiota was shifted by garlic juice; the altered families included Acidaminococcaceae, Akkermansiaceae, Burkholderiaceae, and Erysipelotrichaceae (Fig. 2g). At the genus level, after the garlic juice intervention, the fecal microbiota of high-TMAO producers was comparatively enriched in *Akkermansia*, *Desulfovibrio*, *Christensenellaceae* R − 7 group, and *Lachnospiraceae* UCG − 008 (Supplementary Fig. 7). The volcano plot showed that 12 taxa were significantly altered by the garlic-juice intervention (Fig. 2h). Interestingly, certain beneficial and anti-inflammatory gut commensal bacteria including *Faecalibacterium prausnitzii* and *Akkermansia spp*. were significantly enriched after the 1-week garlic-juice intervention (*P* < 0.05) (Fig. 2i).

### Allicin ameliorated atherosclerosis, reduced TMA/TMAO production, and partially reversed the microbiome shifts in carnitine-treated ApoE^−/−^ mice

**Fig. 3:**
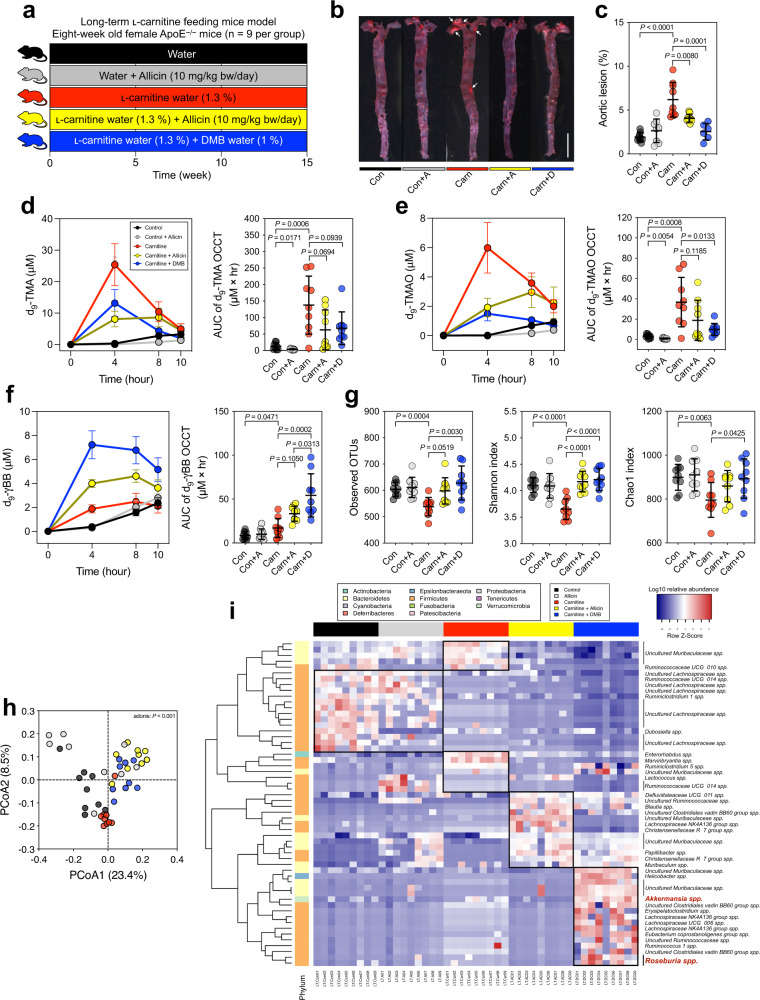
Allicin reduced aortic lesions through the reduction of TMA and TMAO formation and changed the fecal microbiome composition in ʟ-carnitine-induced atherosclerosis female ApoE^−/−^ mice (*n* = 9 per group). **a** experimental design; **b** representative image of oil red-stained enface aorta, scale bar is 0.5 cm; **c** percentage of aortic lesion; **d** d_9_-TMA level according to oral carnitine challenge test (OCCT) and its AUC; **e** d_9_-TMAO level of OCCT and its AUC; **f** d_9_-γBB level according to OCCT and its AUC. **g** α-diversity indices, observed OTUs, Shannon index, and Chao1 index; **h** Principal coordinate analysis (PCoA) plot with Bray−Curtis dissimilarity; and **i** heatmap of the relative abundances of fecal microbiota with a significant difference using the Kruskal–Wallis test with false discovery rate (FDR) (*P* < 0.001). OCCT curves are expressed as the mean ± SEM, and dot plots are expressed as the mean ± SD; Statistical analyses were performed using an unpaired two-tailed Student’s *t*-test Con vs. Con + A group, Con vs. Carn group; one-way ANOVA with Tukey’s range test for comparisons Carn vs. Carn + A group, Carn vs. Carn + D group, and Carn + A vs. Carn + D group.

Our human study demonstrated that raw garlic juice potentially reduced TMAO production and modulated the gut microbiota by enriching beneficial gut bacteria. Thus, we further evaluated the plaque-inhibiting effects of allicin and compared it with DMB in the carnitine-induced atherosclerotic ApoE^−/−^ murine model (Fig. 3a). The ApoE^−/−^ mice fed ʟ-carnitine water showed more advanced aortic plaques than the control group (*P* < 0.0001) (Fig. 3b, c and Supplementary Fig. 8j). Both allicin and DMB significantly reduced aortic plaques (*P* = 0.0080 and *P* < 0.0001). We additionally performed an isotope-labeled d_9_-carnitine challenge test to specifically evaluate the effects of allicin on d_9_-TMA/d_9_-TMAO producing ability from the gut microbiota. The levels of both d_9_-TMA and d_9_-TMAO in the carnitine group were considerably increased, and their area under the curve (AUC) values were significantly higher compared with that in the control group (*P* = 0.0006 and *P* = 0.0008, respectively). Both serum d_9_-TMA and d_9_-TMAO were substantially reduced in the mice of both carnitine + allicin and carnitine + DMB groups compared with the mice fed carnitine alone. Additionally, the control + allicin group showed lower d_9_-TMA and d_9_-TMAO levels than those in the control group (Fig. 3d, e). Although the carnitine + allicin treatment in the ApoE^−/−^ mice showed significantly reduced atherosclerotic plaques compared with carnitine treatment alone, the reduction in TMAO-producing capacity and serum TMAO did not exhibit the expected significant difference. As this is a study model that allicin was administered to ApoE^−/−^ mice over 15 weeks, we speculate that the mouse strain and treatment duration of allicin may modulate the effects of allicin on TMAO reduction. Additionally, d_9_-γBB, an intermediate of d_9_-carnitine metabolism by gut microbiota, increased significantly in the carnitine group compared with that in the control group (*P* = 0.0471) (Fig. 3f). Interestingly, the carnitine + allicin and carnitine + DMB groups showed greater increase in serum d_9_-γBB than those showed by the carnitine group, which is consistent with the result from our previous human study (Fig. 2d) and indicates that the process of d_9_-γBB transformation into downstream TMA might be inhibited, leading to the buildup of d_9_-γBB.

We subsequently investigated the alteration in gut microbiome composition in different treatment groups through 16 S rRNA sequencing analysis with the QIIME pipeline. Regarding the ɑ-diversity, carnitine water alone exhibited decreased observed OTUs, the Shannon index, and the Chao1 index (*P* = 0.0004; *P* < 0.0001; *P* = 0.0063 respectively), whereas additional treatment with allicin or DMB showed favorable ɑ-diversity indices (Fig. 3g). Regarding the β-diversity, the PCoA showed that carnitine water induced a significant microbiome shift (especially at the PCoA2 axis) that was partially reversed by treatment with allicin and DMB (adonis: *P* < 0.001) (Fig. 3h). The heatmap of gut microbial composition showed differences in fecal microbial composition (Fig. 3i); the carnitine + DMB group was enriched in certain beneficial bacteria including *Roseburia* and *Akkermansia*, which correlate inversely with atherosclerotic lesions and protection against atherosclerosis^42,43^. However, in the ʟ-carnitine + allicin group, the relative abundance of these bacteria was lower than that in the ʟ-carnitine + DMB group, suggesting that allicin did not promote the growth of these beneficial bacteria and limited the improvement in atherosclerotic plaques compared with the ʟ-carnitine + DMB group in the carnitine-treated ApoE^−/−^ mice model.

### Raw garlic juice and allicin inhibited microbial carnitine → γBB → TMA pathways in vitro and ex vivo

Our study showed that raw garlic juice reduced the TMAO-producing capacity through carnitine metabolism in TMAO-producing participants, C57BL/6 J mice, and aortic lesion-exhibiting ApoE^−/−^ mice. However, it is unknown whether it can specifically inhibit microbial pathways of carnitine metabolism. Thus, we subsequently performed in vitro and ex vivo studies to examine the inhibitory effects of raw garlic juice and allicin on the following pathway: carnitine → γBB → TMA. In addition, we compared raw garlic juice and allicin with metronidazole, which is a common antibiotic used to treat anaerobic bacterial infections. In functional assays, we used documented type strains responsible for the following pathways: carnitine → γBB and γBB → TMA as an in vitro model and fecal samples from the high-TMAO producers as an ex vivo model.

**Fig. 4:**
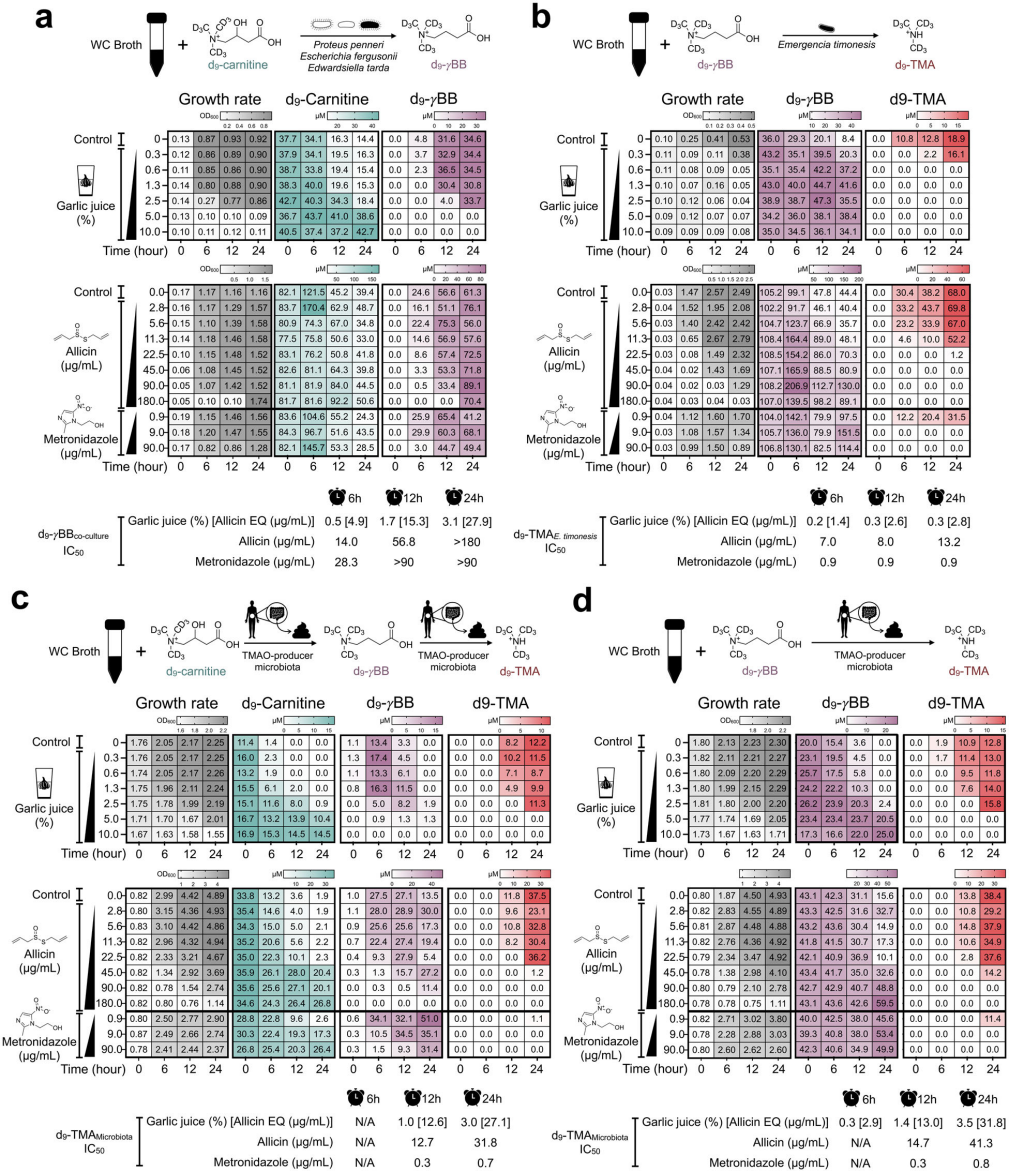
Garlic-juice and allicin inhibited the formation of d_9_-γBB and d_9_-TMA in vitro after inoculation of TMA/γBB-producing bacteria and ex vivo after inoculation of human feces from high-TMAO producers. **a** inhibitory effect of garlic juice and allicin on the bacteria converting carnitine → γBB (co-culture of *Proteus penneri*, *Escherichia fergusonii*, and *Edwardsiella tarda*) in Wilkins–Chalgren (WC) broth supplemented with d_9_-carnitine; **b** inhibitory effect of garlic juice and allicin on the bacteria converting γBB to TMA (*Emergencia timonensis*) in WC broth supplemented with d_9_-γBB. **c** Inhibitory effect of garlic juice and allicin on the high-TMAO-producing gut microbiota in WC broth supplemented with d_9_-carnitine and **d** d_9_-γBB. The utilization of d_9_-carnitine/d_9_-γBB and the production of d_9_-γBB/d_9_-TMA were measured at 0, 6, 12, and 24 h. Half-maximal inhibitory concentration (IC_50_) values were determined using nonlinear regression (see supplementary Fig. 13).

We standardized the carnitine → γBB → TMA pathway functional assay to examine the inhibitory effect of garlic juice and allicin. We established a simplified model by co-culturing type strains that are known to metabolize carnitine to γBB, including *Proteus penneri*, *Escherichia fergusonii*, and *Edwardsiella tarda*. The three co-cultured bacterial strains were inoculated in Wilkins–Chalgren (WC) broth supplemented with d_9_-carnitine. The concentration of garlic juice and allicin was titrated to a potent inhibitory level in the incubation broth. Raw garlic juice at 5% and 10% (45 and 90 µg/mL allicin equivalent (EQ)) exhibited potent inhibition of the transformation of d_9_-carnitine into d_9_-γBB (Fig. 4a). Allicin also was capable of inhibiting the conversion of d_9_-carnitine into d_9_-γBB at 6 and 12 h with half-maximal inhibitory concentration (IC_50_) values of 14.0 and 56.8 µg/mL, respectively. In contrast, the IC_50_ values of garlic juice were 4.9 and 15.3 µg/mL allicin EQ. *E. timonensis* is a bacterium isolated from the human gut; it was recently reported to metabolize γBB into TMA. We observed that raw garlic juice and allicin exhibited potent inhibition of d_9_-TMA production by *E. timonensis* using d_9_-γBB as a substrate at 24 h with IC_50_ equivalent of 2.8 and 13.2 µg/mL, respectively, which is a lower concentration of the prepared garlic juice and allicin (Fig. 4b). The IC_50_ values of garlic juice against the documented strains of bacteria were lower than those of allicin, suggesting that garlic juice may contain other garlic derivative compounds that provided extra inhibitory effect on d_9_-γBB and d_9_-TMA.

To test the inhibitory effect of garlic juice in a complex microbiome community, we collected the fecal microbiota from high-TMAO producing participants and cultured them in WC broth supplemented with d_9_-carnitine or d_9_-γBB. WC broth supplemented with d_9_-carnitine and inoculated with feces from high-TMAO producers showed that d_9_-γBB was produced at 6 h but d_9_-TMA was not detected at that time point. At 12 and 24 h, the d_9_-γBB level was reduced with simultaneous formation of d_9_-TMA. Raw garlic juice at 5% and 10% concentrations (45 and 90 µg/mL allicin EQ) in the culture medium showed optimal inhibition of d_9_-TMA formation with moderate bacterial growth inhibition (Fig. 4c). Allicin at 45, 90, and 180 µg/mL showed potential inhibition of d_9_-TMA produced by TMAO-producer gut microbiota in WC broth supplemented with d_9_-carnitine. Furthermore, the IC_50_ values of both garlic juice and allicin in the ex vivo study were comparable (12 h [12.6 vs. 12.7 µg/mL of allicin]; 24 h [27.1 vs. 31.8 µg/mL of allicin]), suggesting that d_9_-TMA inhibition mainly resulted from allicin in the raw garlic juice. However, compared with metronidazole, allicin exhibited higher IC_50_ value for inhibiting d_9_-TMA production by the gut microbiota, suggesting that metronidazole probably exhibits stronger TMA-reducing activity than allicin. Similarly, conversion of d_9_-γBB into d_9_-TMA started from 6 to 12 h in broth supplemented with d_9_-γBB (Fig. 4d). The d_9_-TMA inhibitory effects of garlic juice and allicin in WC broth supplemented with d_9_-γBB were comparable with those in WC broth supplemented with d_9_-carnitine. Collectively, 3.0–3.5% (27.1–31.8 µg/mL allicin EQ) of garlic juice and 31.8–41.3 µg/mL of allicin were determined to be an d_9_-TMA IC_50_. However, for complete TMA suppression, 5% garlic juice (45 µg/mL allicin EQ) and 90 µg/mL allicin were an optimal concentration for efficiently preventing the utilization of d_9_-carnitine/d_9_-γBB by the fecal microbiota to form d_9_-TMA.

## Discussion

Allicin potentially inhibited the gut microbiota-host-derived TMA and TMAO in C57BL/6 J mice fed with high concentration (1.3%) of ʟ-carnitine, which is consistent with the results of our previous study in which a daily dosage (0.02%) of ʟ-carnitine was administered^40^. In the murine study, ʟ-carnitine intake was the principal factor affecting the overall gut microbiota composition compared with allicin or DMB intervention. The TMAO-reducing effect of allicin is probably based on its broad-spectrum antimicrobial activities^35^. Additionally, previous studies reported that garlic and its bioactive compounds exhibited lipid-lowering and fatty liver-protective effects via gut microbiota modulation^39,44^. Following allicin intake, it can be metabolized to various sulfur-containing bioactive compounds such as diallyl sulfide, diallyl disulfide, and diallyl trisulfide for modulating the microbiome composition in the gut^45–48^.

Previously, in a human study, we developed an OCCT, which can robustly distinguish between low- and high-TMAO producers^41^. In this study, high-TMAO producers were defined as individuals exhibiting OCCT plasma TMAO_MAX_ level ≥ 10 μM^20^. There is currently a lack of studies on the effect of garlic juice on the gut microbiota in the human body; this study demonstrates the effect of garlic juice on TMAO production by the gut microbiota. In this human study, after the intake of garlic juice for 1 week, both plasma and urine TMAO levels were reduced, suggesting that the bioactive compounds in garlic inhibited gut microbial carnitine utilization and TMA-formation ability, resulting in the decrease in TMAO level. The present study demonstrated that plasma and urine TMAO exhibited a high positive correlation that was consistent with the results of previous studies^20,41^. Currently, the primary genes responsible for converting carnitine to TMA by the gut microbiota remain unclear. A Rieske-type microbial *CntA/B* enzyme was reported to convert carnitine to TMA^15^. However, it is doubtful whether *CntA/B* is responsible for TMA formation in the gut anaerobic environment because oxygen is required for its reaction^18,41^. A recent study showed that ʟ-carnitine can be metabolized to the intermediate γBB, followed by transformation into TMA via a less abundant anaerobic bacterium called *E. timonensis*; this could be the major pathway for TMA generation by the gut microbiota in the oxygen-limited environment^49^. This study showed an increase in plasma γBB following the garlic-juice intervention, suggesting that garlic juice might block the conversion of γBB to TMA, resulting in accumulation of γBB. The γBB level was increased in both human and ApoE^−/−^ mice administered with raw garlic juice or allicin but decreased in 2-week allicin-fed C57BL/6 J mice. The difference in γBB level could be caused by different mouse strains comprising distinct gut microbiota composition. In fact, both ɑ- and β-diversity of the fecal microbiota of C57BL/6 J and ApoE^−/−^ mice were different (Supplementary Fig. 9a–d). In addition, the strain effect was more prominent than the treatment effect (Supplementary Fig. 9e, f).

The gut microbiome analysis showed that the garlic-juice intervention increased the Shannon index, indicating that a more diverse and balanced microbial community was produced by garlic-juice consumption. The overall gut microbiome profile for each individual was moderately affected by the garlic-juice intervention; however, the relative abundance of certain beneficial gut bacteria was increased following the garlic-juice intake, including *Akkermansia* and *Faecalibacterium prausnitzii*. A recent study also reported that allicin treatment in high-fat diet mice caused the enrichment of beneficial bacteria such as *Bifidobacterium* and *Lactobacillus*^50^. *Akkermansia muciniphila* has been proved to improve the metabolic profile and reduce metabolic disorders, obesity, endotoxemia, adipose tissue inflammation, and insulin resistance^51^. An extracellular vesicle and a glucagon-like peptide-1-inducing protein from *A. muciniphila* improve glucose homeostasis and metabolic disease in mice^52,53^. Furthermore, it shields against atherosclerosis by blocking metabolic endotoxemia-induced inflammation in ApoE^−/−^ mice^43^. The garlic intervention increased the abundance of *Faecalibacterium prausnitzii*, which has been reported to be an anti-inflammatory commensal bacterium that secretes a metabolite blocking NF-κB activation and IL-8 secretion^54^. These results suggested that the garlic-juice intervention modulates the gut microbiota of high-TMAO producers in a favorable direction. However, drinking raw garlic juice with an empty stomach may cause abdominal discomfort, and thus it should be consumed with a meal to prevent undesirable stomachache.

In ApoE^−/−^ mice, 1.3% ʟ-carnitine-induced atherosclerosis, that was ameliorated by allicin supplementation, indicating the potential anti-atherosclerotic activity of allicin. However, we observed that TMAO reduction by allicin was more effective with short-term treatment in C57BL/6 J mice than with a long-term treatment in ApoE^−/−^ mice, suggesting that long-term allicin administration could result in the resistance of TMA-producing bacteria against the antimicrobial effect of allicin. The allicin, DMB, and ʟ-carnitine groups in the short-term study C57BL/6 J mice exhibited a similar gut microbiota composition. However, long-term allicin administration resulted in more differences in the overall gut microbial composition of ApoE^−/−^ mice. We speculated allicin may require a longer time to modify the overall gut microbial shift in the C57BL/6 J mice study. As the mice of each group were housed in separate cages and each group was divided into three cages, we observed the cage effect on gut microbial shift. However, the treatment effects on the gut microbiota were more prominent than the cage effect (Supplementary Fig. 10). Additionally, in vitro and ex vivo studies suggested that both garlic and allicin inhibited the formation of both d_9_-γBB and d_9_-TMA in the culture medium inoculated with the co-culture of bacteria producing γBB (*P. penneri*, *E. fergusonii*, and *E. tarda*) and TMA (*E. timonensis*), as well as inhibited TMA formation by the complex bacterial consortium of the human TMAO-producing fecal microbiota. Although metronidazole has a lower TMA IC_50_ than allicin and garlic juice in the ex vivo study, it does not necessarily suggest that a broad-spectrum antibiotic is more appropriate than a garlic-based regimen in practice. In fact, the garlic intake equivalent to the experimental TMA-inhibitory dose of garlic juice is potentially achieved from dietary intake, whereas chronic consumption of broad-spectrum antibiotics for the TMA inhibition may not be advisable. The concentration of allicin and other bioactive compounds from garlic can be even higher surrounding the substrates when raw garlic is chewed and consumed together with carnitine-rich foods (such as red meat), which may form an antimicrobial coating on carnitine-rich foods and further prevent gut bacteria from utilizing carnitine to produce TMA. Moreover, garlic may provide additional benefits by favorably shaping the gut microbial community, which allows it to be a more plausible option than broad-spectrum antibiotics in current practice.

**Fig. 5:**
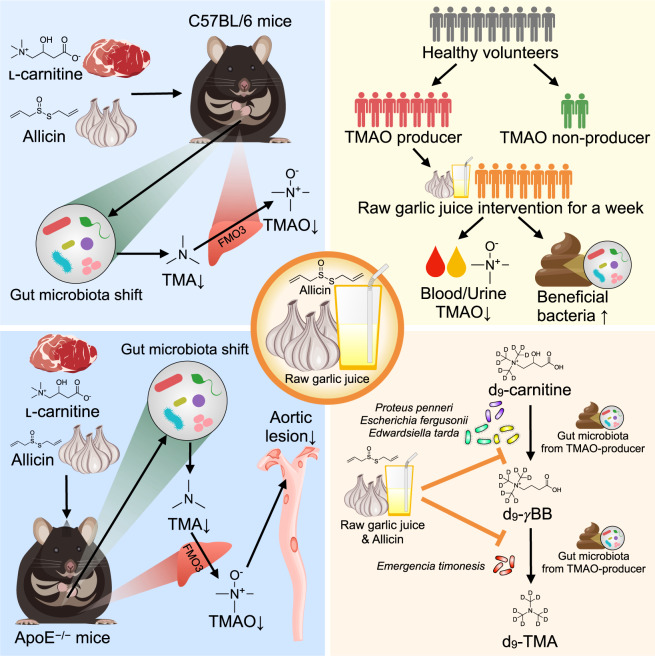
Effect of allicin and raw garlic juice on CVD prevention and atherosclerosis amelioration through gut microbiota and TMAO modulation. (1) Allicin decreased the gut microbiota- and host-derived TMA and TMAO levels in mice supplemented with ʟ-carnitine in drinking water; (2) Raw garlic juice reduced the plasma and urine TMAO levels and increased the beneficial gut bacterial abundance in high-TMAO producing humans; (3) Allicin ameliorated aortic lesions by reducing the TMA/TMAO production and altered the gut microbiome in ʟ-carnitine-induced atherosclerotic ApoE^−/−^ mice; (4) Raw garlic juice and allicin inhibited microbial pathway of carnitine → γBB → TMA conversion in specific bacteria and high-TMAO producer’s gut microbiota.

Here, we demonstrated that supplementation with allicin or raw garlic juice inhibited TMAO production through carnitine metabolism by the gut microbiota in both mice and humans and prevented carnitine-induced atherosclerosis in ApoE^−/−^ mice. This was supported by in vitro and ex vivo assays using specific bacteria and the high-TMAO producer feces that metabolize ʟ-carnitine to γBB and γBB to TMA (Fig. 5). These data provide valuable evidence that raw garlic, which contains allicin, shifts the gut microbiota composition and modulates the following gut microbial pathway: ʟ-carnitine → γBB → TMA. In summary, this study suggests that garlic might serve as a potential dietary intervention for CVD prevention.

## Methods

### Allicin preparation for the animal study

Information on the resources is provided in Supplementary Table 2. Allicin synthesis, purification, and identification were based on our laboratory protocol^39,40,55^. The purity of allicin was >97%; it was dissolved in 0.5% carboxymethyl cellulose (CMC) and stored at −80 °C before administration to the mice.

### ʟ-carnitine-fed C57BL/6 J mouse model

The handling of animals complied with the guidelines of the Institutional Animal Care and Use Committee of National Taiwan University (approval number: NTU107-EL-00170 and NTU107-EL-00084). Six-week-old male C57BL/6 J mice were purchased from Taiwan National Laboratory Animal Center. The mice were housed in a room with a 12 h light–dark cycle at 22 ± 2 °C after an adaptation period of 2 weeks. The mice were randomly divided into experimental groups (i) control; (ii) control + allicin; (iii) carnitine; (iv) carnitine + allicin; and (v) carnitine + DMB. Mice for each treatment were housed in separate cages and each treatment was divided into three cages (*n* = 2–4 per cage). For the carnitine intake group, 1.3% ʟ-carnitine in water was supplied^10^. Allicin treatment groups were orally administered 10 mg/kg bw allicin in 0.5% CMC per day^40^. The DMB group was treated with 1% DMB in water^24^. The mice were sacrificed by CO_2_ asphyxiation after 2 weeks. The blood was collected by cardiac puncture using a syringe.

### Long-term ʟ-carnitine-induced atherosclerosis ApoE^−/−^ mouse model

The ApoE^−/−^ mice were initially purchased from the Jackson Laboratory (Bar Harbor, ME) and bred in our laboratory animal room according to the guidelines of the Institutional Animal Care and Use Committee of National Taiwan University. Six-week-old female ApoE^−/−^ mice were used for the experiment after an adaptation period of 2 weeks. Female ApoE^−/−^ mice were used in this study because of a more prominent flavine monooxygenase 3 (FMO3) activity and a notable increase in plasma TMAO than those in male ApoE^−/−^ mice according to a previous report^56,57^. The mice were grouped, treated, and housed similar to the short-term C57BL/6 J mouse model, as described previously. The ApoE^−/−^ mice were sacrificed after 15 weeks. The blood was collected by cardiac puncture using a syringe. Phosphate-buffered saline (PBS) was used to gently flush the aorta to remove blood clots, and the aorta was removed carefully from the body.

### Oral d_9_-carnitine challenge test in the mouse

On the day of the sacrifice, the mice were gavaged with 200 μL (150 mM) of d_9_-carnitine, followed by serial blood collection at 0, 4, 8, and 10 h^40^. Subsequently, the mice were subject to carbon dioxide asphyxiation. The blood was collected by cardiac puncture using a syringe at 12 h. The blood samples were centrifuged at 1000 × *g* for 15 min. The supernatants were collected and stored at −80 °C before further analysis.

### Oil red staining of the aorta

Aortic specimens were rinsed with PBS to remove excess blood residue. Dissection was performed under the microscope using micro-dissection scissors and forceps to remove the adipose tissue. The aorta was fixed with 10% formalin and stained with oil red O dye^58^ as follows: it was (1) washed with deionized water for 5 min; (2) soaked in propylene glycol for 10 min; (3) stained with oil red O dye; (4) soaked in 85% propylene glycol for 3 min; and (5) washed twice with deionized water. We captured images of the stained aorta and analyzed the aortic lesion area using the Image J software (Version 1.8.0).

### Mouse blood biochemistry analysis

The serum was extracted by centrifuging the blood at 1000 × *g* for 15 min at 4 °C. We analyzed serum biochemical parameters including total cholesterol, total triglyceride, high-density lipoprotein (HDL-c), AST, and ALT using commercial test strips (Spotchem II reagent strips; Arkray Inc., Kyoto, Japan) in an automatic blood analyzer (Spotchem EZ)^59^.

### High-TMAO producer screening by OCCT

This research was approved by the Research Ethics Committee of National Taiwan University Hospital (201712031RIND), and the study has been registered on ClinicalTrials.gov as NCT04545879. Informed consent was obtained from all study participants. Healthy participants were recruited (*n* = 9), under the following criteria: (1) age ≥ 20 years old; (2) no exposure to antibiotics, probiotics, or carnitine supplements within the previous month; (3) no history of chronic diseases including diabetes mellitus, myasthenia gravis, chronic renal disease, hyperparathyroidism, epilepsy, and severe anemia; (4) participants were excluded from the study if they reported recent gastrointestinal discomfort (such as abdominal pain or diarrhea).

We used the OCCT, which was previously shown to exhibit better efficacy than fasting plasma TMAO, to identify the TMAO producer phenotype^41^. All participants fasted at least 8 h before the OCCT. The participants were orally administered 1500 mg of ʟ-carnitine (3 tablets, General Nutrition Centers, Inc., USA). The blood and urine of the participants were collected at 0, 24, 48, and 72 h after carnitine intake. The plasma and urine samples were centrifuged at 1000 × *g* for 15 min and stored at −80 °C before the following analyses. Participants with plasma TMAO_MAX_ ≥ 10 μM after the OCCT were defined as high-TMAO producers and allowed to proceed for the garlic juice intervention^20^.

### Garlic juice preparation and allicin content quantification

Raw garlic was peeled to remove the skin; 100 g of garlic and 300 mL of water (ratio 1:3) were mixed and blended into garlic juice using a blender. After filtration, the raw garlic juice was placed into a glass bottle (55 mL for each), ready to be consumed by the subjects. The garlic juice required was prepared at once and frozen at −20 °C before use.

The allicin content in the raw garlic juice was determined using HPLC (JASCO LC-NetII/ADC/JASCO UV-2075 Plus) with Shiseido C18 (5 µm, 4.6 × 250 mm). The mobile phase was deionized water and methanol, and the flow rate was 1 mL/min. The gradient program was run as follows: 0–100% methanol, 0–30 min, and 100% methanol, 10 min. The absorbance was detected at 254 nm. The allicin concentration in raw garlic juice was calculated against the allicin standard curve at concentrations of 0.2, 0.4, 0.6, 0.8, and 1.0 mg/mL.

### Garlic-juice intervention

High-TMAO producers (*n* = 7) were asked to consume 55 mL of raw garlic juice (48 mg of allicin equivalent) once a day during dinner for 1 week. Participants were suggested to consume the garlic juice with a meal to decrease stomach irritation caused by the quick drinking of raw garlic juice. The high-TMAO producers were free to choose their diet with no restriction on food type. After 1 week of raw garlic juice intervention, the second OCCT was performed.

### Gut microbiota inoculum preparation from human feces for ex vivo study

One gram of raw fecal sample was mixed with 10 mL of PBS supplement with 0.05% ʟ-cysteine in a tube containing glass beads, homogenized the mixture by vortexing for 1 min in an anaerobic chamber (Whitley DG250 Workstation, Don Whitley Scientific Limited, UK), filtered the homogenized fecal liquid through a Falcon^®^ 100 µm cell strainer, and subsequently used the fecal filtrate for the study.

### Media selection study for maximal TMA production

To investigate the suitability of the culturing media for producing maximal TMA concentration, we compared three media: gut microbiota medium (GMM)^60^, MEGA^61^, and WC medium^62^. Furthermore, we modified these media as a carbon-reduced medium. The media were supplemented with 100 μM ʟ-carnitine. The media recipes are provided in Supplementary Tables 3–5. Two gut microbiota inocula from the feces of high-TMAO producers were prepared by mixing 1 g of feces with 15 mL of PBS with 0.05% cysteine and filtering the mixture through a 100-μm Falcon cell strainer, following which 100 μL of the stool filtrate was transferred to 1.9 mL of medium and subsequently incubated at 37 °C under anaerobic conditions (80% N_2_, 10% CO_2_, and 10% H_2_) for 0, 6, 12, and 24 h. The cultures were collected, their growth measured at optical density (OD) of 600 nm (Spectronic Helios Gamma UV-Vis Spectrophotometer, Thermo Fisher Scientific, UK), centrifuged at 3500 × *g* for 1 min, and the supernatant was collected for measuring TMA, TMAO, γBB, carnitine, and choline concentration using LC-MS.

### Gut microbiota inoculum and bacteria

The medium used in this study was WC broth supplemented with d_9_-carnitine or d_9_-γBB. We used the WC broth because it produces the highest TMA level among GMM, MEGA medium, and its carbon-reduced medium (Supplementary Fig. 11). The high-TMAO producer feces for the inhibition study was re-collected from the participant (GJ07), which exhibited the highest TMAO_MAX_ during the OCCT and possessed the *E. timonensis* 16 S sequence in the sample. The bacteria used in this study included the bacteria converting ʟ-carnitine to γBB, including *P. penneri* ATCC33519, *E. fergusonii* ATCC35469, and *E. tarda* ATCC15947^63^, and *E. timonensis* DSM101844 which transforms γBB to TMA^18^. A single colony on the WC agar plate of the bacteria was inoculated into the WC broth for 24 h under anaerobic conditions before being used in the inhibition study (Supplementary Fig. 12).

### Inhibition study of raw garlic juice and allicin against high-TMAO producer gut microbiome and γBB/TMA-producing bacteria

Garlic juice was prepared as described above, following which it was sterilized by filtration through 0.22-μm polyvinylidene fluoride membranes. We inoculated 50 μL of the gut microbiota inoculum from the high-TMAO producers, which was prepared as described above, in 1 mL of WC broth with 50 μM d_9_-carnitine or d_9_-γBB. Subsequently, the sterilized raw garlic juice was added at final concentrations of 0, 0.3, 0.6, 1.3, 2.5, 5.0, and 10.0%; allicin at 0, 2.8, 5.6, 11.3, 22.5, 45.0, 90.0, and 180.0 µg/mL; metronidazole at 0.9, 9.0, and 90.0 µg/mL. Then, incubated at 37 °C under anaerobic conditions (80% N_2_, 10% CO_2_, and 10% H_2_) for 0, 6, 12, and 24 h. We collected the cultures, estimated the growth OD_600_, centrifuged the cultures at 3500 × *g* for 1 min, and collected the supernatant for measuring d_9_-TMA, d_9_-γBB, and d_9_-carnitine using LC-MS. For evaluating the inhibition of bacteria related to CVD, the γBB-producing bacteria *P. penneri*, *E. fergusonii*, and *E. tarda* were mixed before the inhibition analysis. We inoculated 100 μL of the γBB-producing bacterial mixture in 1 mL of WC broth with 100 μM d_9_-carnitine, added garlic juice, cultured the bacteria, and performed the analysis as mentioned above. For analyzing the inhibition of *E. timonensis*, we inoculated 100 μL of the inoculum in 1 mL of WC broth with 100 μM d_9_-γBB, added garlic juice/allicin/metronidazole and cultured the bacteria, and performed the analysis as mentioned above.

### Measurement of TMA, d_9_-TMA, TMAO, d_9_-TMAO, γBB, d_9_-γBB, carnitine, and d_9_-carnitine levels in mice by LC-MS

For sample preparation, 50 μL of mouse serum was added to 450 μL of methanol containing isotopically labeled internal standards (d_3_-carnitine, d_9_-TMAO, and ^13^C_3_-TMAO). Subsequently, the mixture was centrifuged at 12,000 × *g*, 4 °C for 5 min, and the supernatants were collected for LC-MS.

For LC-MS analysis, the target metabolites of the serum samples were analyzed using the Agilent 1290 UHPLC tandem Agilent 6460 triple quadrupole mass spectrometer. The MicroSolv Cogent Diamond Hydride column (150 × 2.1 mm, 4.2 μm, MicroSolv, Eatontown, NJ) was used in this study by maintaining the column temperature at 40 °C. Mobile phase solution A was 10 mM ammonium acetate, 0.2% formic acid in deionized water; solution B was 10 mM ammonium acetate and 0.2% formic acid in 90% acetonitrile. The flow rate was 0.4 mL/min, and the gradient program was as follows: 90–75% solution B for 0–1 min; 75–65% solution B for 1–2 min; 65–55% solution B for 2–4 min; 55–40% solution B in 4–5 min, followed by re-equilibration of the column with 90% solution B for 1 min.

For MS, the positive electrospray ionization mode was used with the following parameters: drying gas temperature was set at 325 °C, flow rate 7 L/min, nebulizer pressure at 45 psi, sheath gas temperature at 325 °C, 11 L/min sheath gas of flow rate, the capillary voltage at 3500 V, and nozzle voltage at 500 V. The mass spectrometer was configured in multiple reaction monitoring modes. The monitored transitions for carnitine were: m/z 162.1 → 43.2 and 162.1 → 60.2; d_3_-carnitine, m/z 165.1 → 43.1 and 165.1 → 61.2; TMAO, m/z 76.1 → 58.1 and 76.1 → 59.1; d_9_-TMAO, m/z 85.1 → 66.3 and 85.1 → 68.3; ^13^C_3_-TMAO, m/z79.1 → 61.1 and 79.1 → 62.1; TMA, m/z 60.1 → 45.2 and 60.1 → 44.1; d_9_-TMA, m/z 69.1 → 51.1 and 69.1 → 49.1; and ^13^C_3_-TMA, m/z 63.1 → 46.1. The detected peak area ratio was used to calculate the concentration of each target analyte in the serum sample against the calibration curve.

### Measurement of TMA, d_9_-TMA, TMAO, d_9_-TMAO, γBB, d_9_-γBB, carnitine, d_9_-carnitine, and choline levels in human and culture medium by LC-MS

For sample preparation, 1 μL of plasma, urine, or culture medium was mixed with 199 μL of isotopically labeled internal standards (d_3_-carnitine, d_9_-TMAO, and ^13^C_3_-TMAO) in 0.1% formic acid acetonitrile solution. The solution was subsequently centrifuged at 20,000 × *g*, 0 °C for 5 min. For urine sample preparation, the urine was centrifuged at 3000 × *g*, 22 °C for 15 min. We mixed 5 μL of the 1st supernatant obtained with 45 μL of isotopically labeled internal standards (d_3_-carnitine, d_9_-TMAO, and ^13^C_3_-TMAO) in 0.1% formic acid acetonitrile solution. The solution was centrifuged at 20,000 × *g*, 0 °C for 5 min, and the 2nd supernatant was obtained. Next, 2 μL of the 2nd supernatant was mixed with 198 μL of internal standard and centrifuged at 20,000 × *g*, 0 °C for 5 min; the supernatant obtained was ready for LC-MS/MS quantification. The prepared plasma and urine samples were analyzed using LC-MS/MS SCIEX 5500 for TMA, TMAO, γBB, carnitine, and choline quantification.

For LC-MS/MS analysis, 20 μL of each plasma or urine sample was injected into a Sciex Exion LC AC system coupled with a SCIEX Triple TOF 5600 mass spectrometer (AB SCIEX, Canada). The separation was performed using a HILIC column (250 × 4.0 mm, 5 μm, Fortis, UK) maintained at 40 °C. Mobile phase A was 0.1% formic acid in deionized water, and mobile phase B was 0.1% formic acid in acetonitrile. The flow rate was 0.5 mL/min. The LC program: 0–1 min, 50% solvent B, 1–9 min, 50–40% solvent B, 9–10 min, 40% solvent B, 10–10.1 min, and 40–50% solvent B, followed by column re-equilibration with 50% solvent B for 1.9 min. The electrospray was set in positive ionization mode with the following parameters: curtain gas supply, 30 psi; capillary temperature, 500 °C; spray voltage floating, 5500 V; and declustering potential, 80 V. The concentration of each analyte was calculated from calibration curves relating the peak area ratio to its corresponding standard.

### Platelet preparation and aggregometry assays

Platelet preparation and aggregometry assays were performed according to the methods used by Zhu et al. (2016) with modifications^16^. Whole blood was collected from study participants using a 3.2% sodium citrate anticoagulant. Platelet-rich plasma (PRP) was obtained by centrifugation at 100 × *g*, 22 °C for 6 min. Platelet-poor plasma (PPP) was prepared by further centrifugation at 11,000 × *g* for 2 min. Platelets were counted using Sysmex K1000 Hematology Analyzer, and the CHRONO-LOG490-4D system was used for aggregometry assays. Platelet concentrations were adjusted to 2 × 108/mL with PPP. 5 μM ADP was used as indicated to initiate aggregation with constant stirring (600 rpm). Both PRP and PPP samples were kept at 37 °C before the aggregometry assays were performed.

### Fecal DNA extraction and 16 S rRNA amplicon sequencing and sequencing analysis

Fecal sample collection and genomic DNA extraction: Mouse fecal samples were collected from the large intestine during a sacrifice from both the long-term study in ʟ-carnitine-induced ApoE^−/−^ mice and short-term study in the C57BL/6 J experimental mouse model; the samples were snap-frozen using liquid nitrogen and stored at −80 °C before use. Human fecal samples were collected before and after the garlic juice intervention using a stool collection kit^64^. The samples were stored at −80 °C for further examination. Fecal genomic DNA was extracted using the QIAmp Power Fecal DNA Kit (QIAGEN, Netherlands) according to the manufacturer’s instructions and quantified using the NanoDrop ND-1000 spectrophotometer (Thermo Fischer Scientific).

Polymerase chain reaction (PCR), library preparation, and 16 S rRNA gene sequencing: The V3-V4 hypervariable region of the 16 S rRNA gene was amplified using the primer pair ((Forward = 5′-TCG TCG GCA GCG TCA GAT GTG TAT AAG AGA CAG CCT ACG GGN GGC WGC AG-3′) and (Reverse = 5′-GTC TCG TGG GCT CGG AGA TGT GTA TAA GAG ACA GGA CTA CHV GGG TAT CTA ATC C-3′)). PCR amplification was performed in a 25 μL reaction mixture containing 5 ng of DNA template, 0.2 μM forward and reverse primers, and 12.5 μL of 2× Taq Master Mix (KAPA HiFi HotStart ReadyMix, Roche, Switzerland). The PCR conditions involved an initial step at 95 °C for 3 min, followed by 25 cycles of 95 °C for 30 s, 55 °C for 30 s, 72 °C for 30 s, and a final extension of 72 °C for 5 min. Subsequently, 2% agarose gel electrophoresis was used to visualize the amplified products. Dual index and Illumina sequencing adapters were attached by using a Nextera XT Index Kit via PCR according to the manufacturer’s instructions. PCR product cleanup was conducted using AMPure XP beads to purify the V3-V4 amplicon. The sizes of PCR products were confirmed using the Bioanalyzer DNA 1000 chip. Library quantification was conducted for quality control before sequencing using the Agilent Technologies 2100 Bioanalyzer. The pooled libraries were subjected to paired-end sequencing (2 × 300 bps) using the Illumina MiSeq platform.

Sequencing analysis: The raw sequences were processed following the instructions in the 16 S Bacteria/Archaea SOP v1 of the Microbiome Helper workflows (https://github.com/mlangill/microbiome_helper)^65^. The paired-end reads were filtered as a sequence length of over 400 and a quality score of 90% at a Phred score of 30, followed by the removal of the chimeric sequences using VSEARCH v2.1.2^66^. The high-quality reads were subsequently analyzed using QIIME v.1.9.1^67^. OTUs were produced using the UCLUST algorithm and a closed-referenced OTU approach against the SILVA database (version 132) with 97% of sequence identity, followed by rarefaction. The vegan package in R was used to calculate α-diversity indices, including the observed OTUs, Shannon index, and Chao1 index, PCoA based on the Bray–Curtis distance. We performed permutation multivariate analysis of variance (ANOVA) using distance matrices (adonis) to determine the heterogeneity of the fecal microbiota among the groups. A heatmap was plotted using the heatmap3 package.

### Statistical analysis

Statistical analysis was selected on the basis of whether the datasets were normally distributed or not. The animal or participant number (*n*) is stated in the figure legend, and each dot in the plot represents each mouse or participant. For the animal study, the data are represented as the mean ± standard error of the mean (SEM) or mean ± standard deviation (SD) or median. Parametric tests (unpaired two-tailed Student’s *t*-test or one-way ANOVA with Tukey’s range test) and non-parametric tests (Wilcoxon signed-rank test or The Kruskal–Wallis test with Dunn’s multiple comparison test) were performed to compare the differences in the animal study. The Student’s *t*-test or Wilcoxon signed-rank test was performed (Con vs. Con + A; Con vs. Carn). One-way ANOVA with Tukey’s range test or the Kruskal–Wallis with Dunn’s multiple comparison test was performed for the one variable with three levels (Carn vs. Carn + A; Carn vs. Carn + D; Carn + A vs. Carn + D). For the human study, data are expressed as the mean ± SEM; paired two-tailed Student’s *t*-test was performed to compare the difference in data between before and after garlic juice intake. The Kruskal–Wallis test, Wilcoxon signed-rank test with or without false discovery rate, unpaired/paired two-tailed Student’s *t*-test, and one-way ANOVA with Tukey’s range test were performed for the analysis of fecal microbial data based on the dataset distribution. Permutational Multivariate Analysis of Variance Using Distance Matrices (PERMANOVA; adonis) was performed to assess the significance of between-group differentiation based on the Bray–Curtis dissimilarity. All statistical analyses were performed using Graphpad Prism (version 9.2.0), R (version 3.6.1), or R Studio (version 1.2.5001).

### Reporting summary

Further information on research design is available in the Nature Research Reporting Summary linked to this article.

## Supplementary information

Supplementary information

Reporting Summary

## Data Availability

The raw 16 S rRNA sequencing data used to produce all figures are accessible at the NCBI Short Read Archive under the following accession numbers: BioProject: PRJNA661156 and SRA: SRS7316558, SRS7317051, and SRS7315455.

## Code availability

Bioinformatic tools, software version, parameters, and open-source code used in this present study are described in the “Methods” section. More details regarding the code to reproduce the analyses are available upon request.
