# Supplementary material for: Atherosclerosis amelioration by allicin in raw garlic through gut microbiota and trimethylamine-N-oxide modulation
Source: NPJ Biofilms Microbiomes. 2022 Jan 27;8:4. doi: 10.1038/s41522-022-00266-3 (PMC8795425; doi:10.1038/s41522-022-00266-3)
Supplement: Supplementary file 1 — Supplementary information [file 41522_2022_266_MOESM1_ESM.pdf]

Supplementary Information

Atherosclerosis Amelioration by Allicin in Raw Garlic through Gut Microbiota and Trimethylamine-*N*-Oxide Modulation

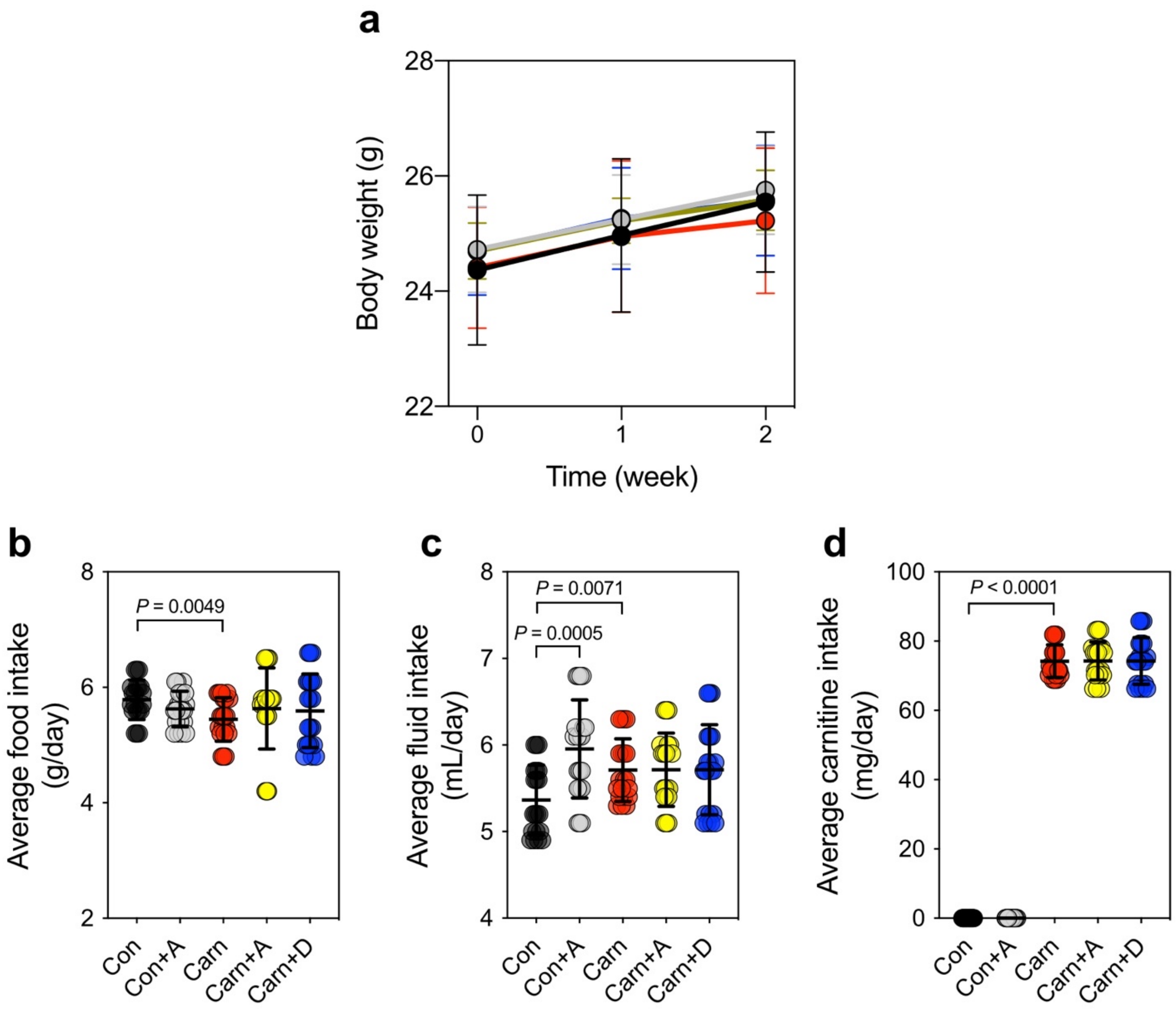

Supplementary Fig. 1 | Effect of allicin and carnitine on L-carnitine-induced atherosclerosis in male C57BL/6J mice. (a) Changes in body weight; (b) average food intake; (c) average fluid intake; and (d) average carnitine intake. Dot plots are expressed as the mean ± SD. Statistical analyses were performed using the unpaired two-tailed Student's t-test Con vs. Con + A group, Con vs. Carn group; one-way ANOVA with Tukey's range test for comparisons Carn vs. Carn + A group, Carn vs. Carn + D group, and Carn + A vs. Carn + D group.

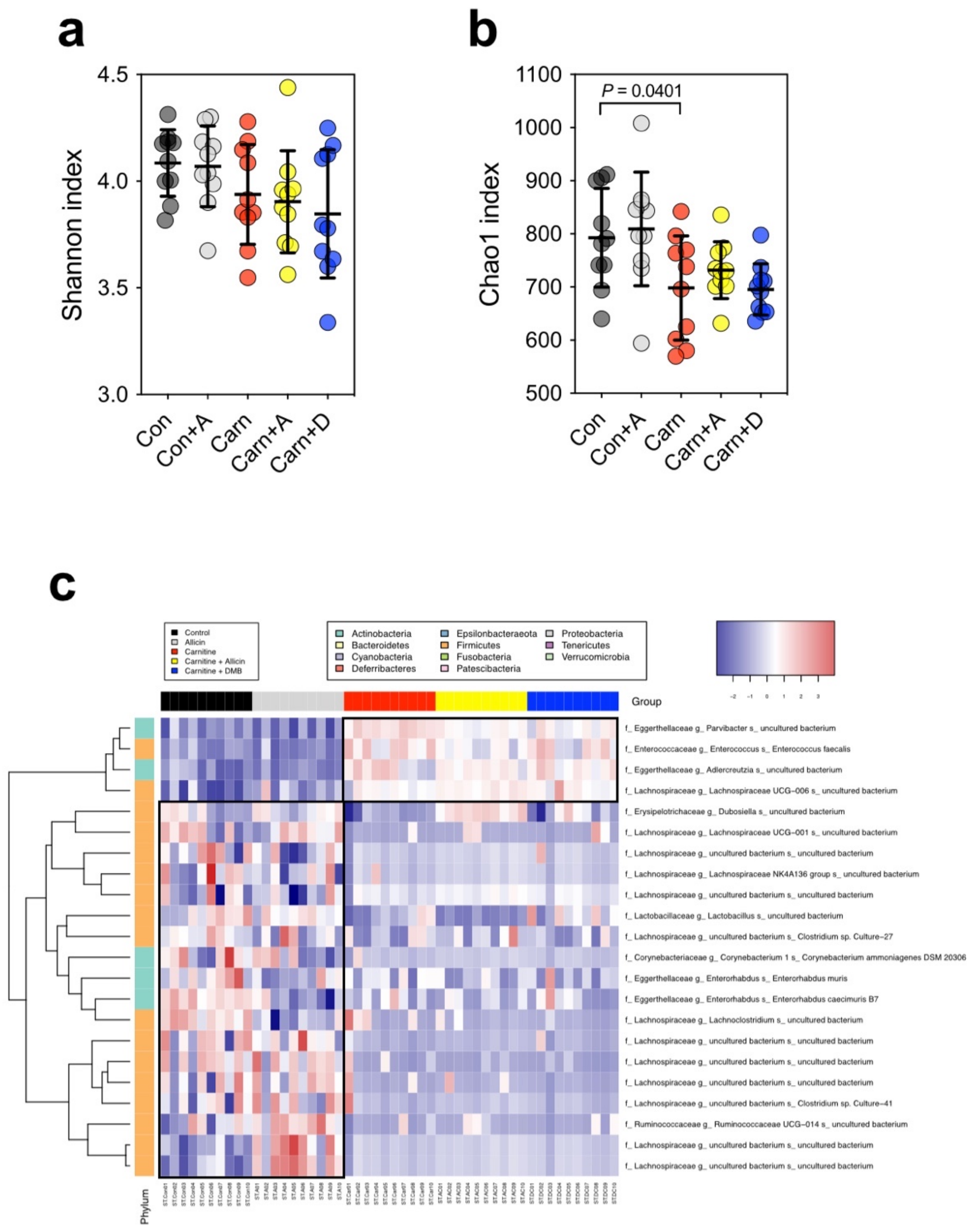

Supplementary Fig. 2 | ʟ-carnitine primarily affected the fecal microbial composition of male C57BL/6J mice. α-diversity indices, (a) Shannon index and (b) Chao1 index. (c) Heat map of the relative abundances at OTU level of fecal microbiota with a significant difference, as evaluated using the Kruskal–Wallis test with false discovery rate (FDR) (q < 0.01). Dot plots are expressed as the mean ± SD. Statistical analyses of α-diversity indices were performed using the unpaired two-tailed Student's t-test Con vs. Con + A group, Con vs. Carn group; one-way ANOVA with Tukey's range test for comparisons Carn vs. Carn + A group, Carn vs. Carn + D group, and Carn + A vs. Carn + D group.

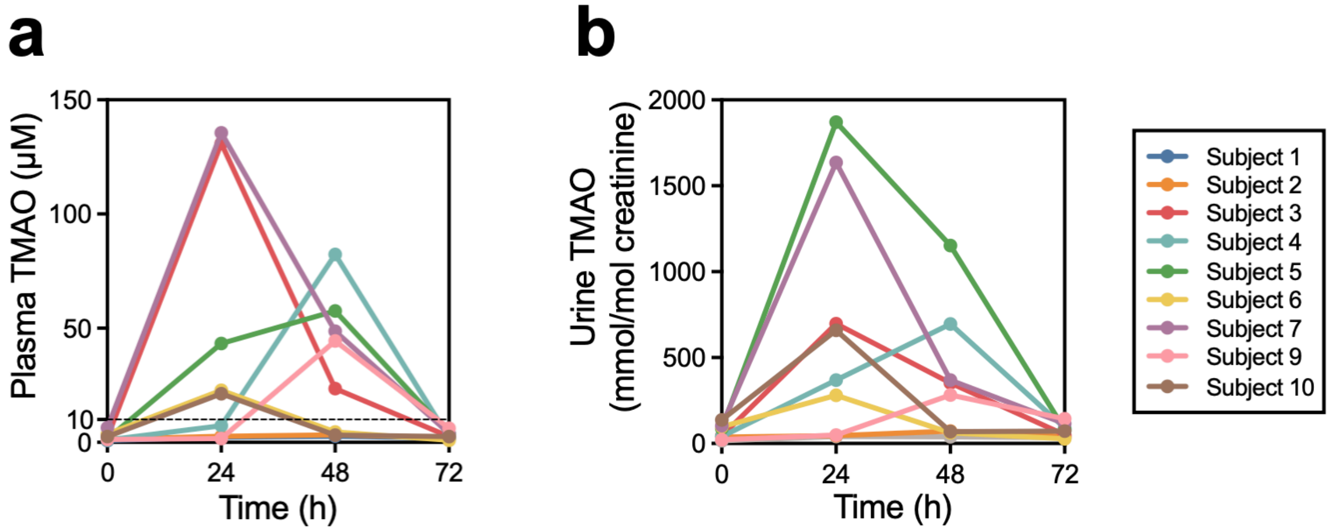

Supplementary Fig. 3 | Screening of high-TMAO producers using oral carnitine challenge test (OCCT) based on plasma TMAO level ≥ 10 μM in participants (n=9). (a) Plasma and (b) urine TMAO; OCCT was performed by collecting the blood and urine samples as a baseline and subsequently administering three carnitine tablets (1,500 mg), followed by the collection of blood/urine at 24, 48, and 72 h.

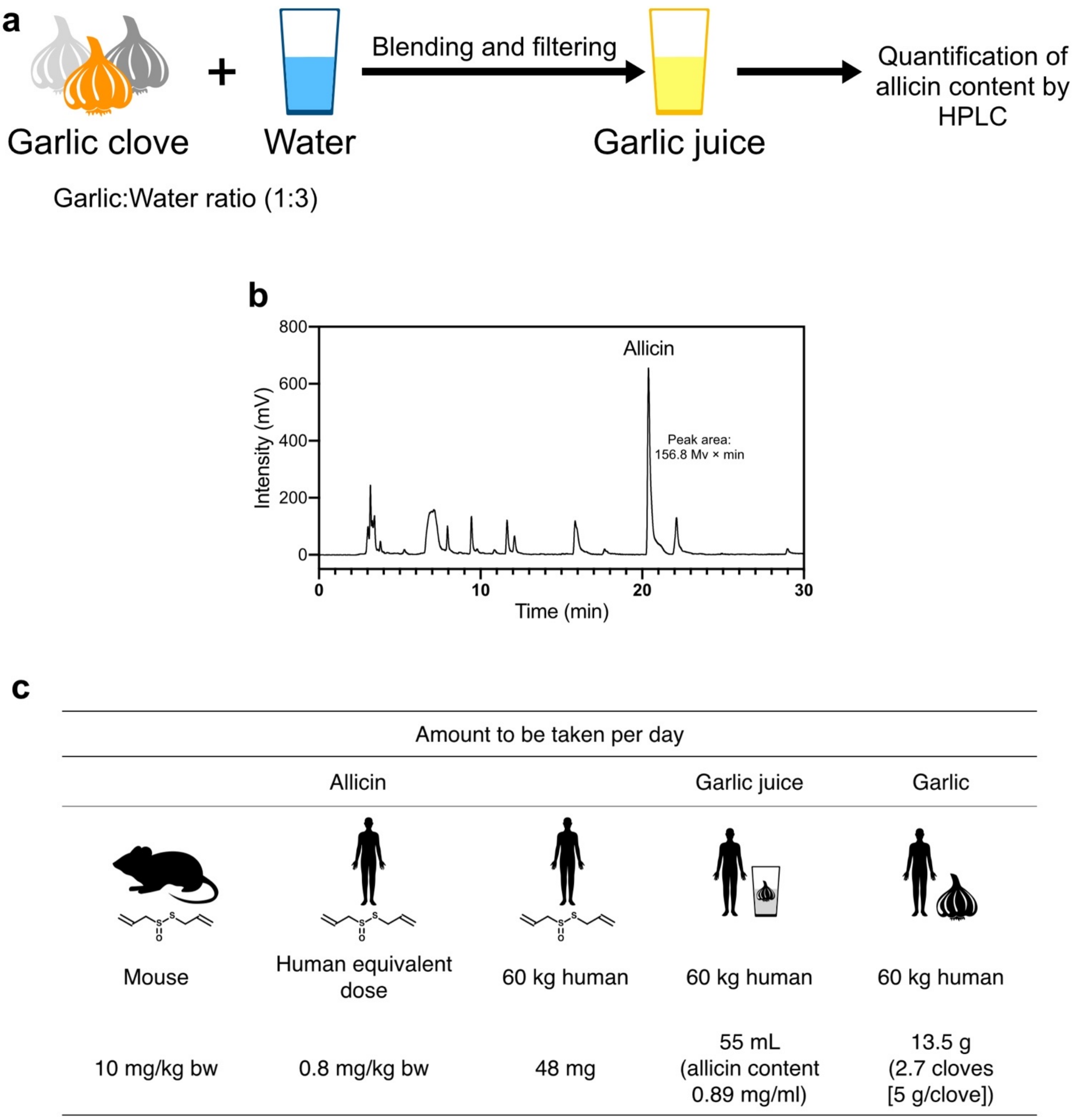

Supplementary Fig. 4 | Garlic juice preparation and mouse to human equivalent dose translation. (a) Garlic juice preparation; (b) HPLC chromatogram indicates that the major component in garlic juice is allicin; (c) mouse to human equivalent dose translation of allicin and garlic juice.

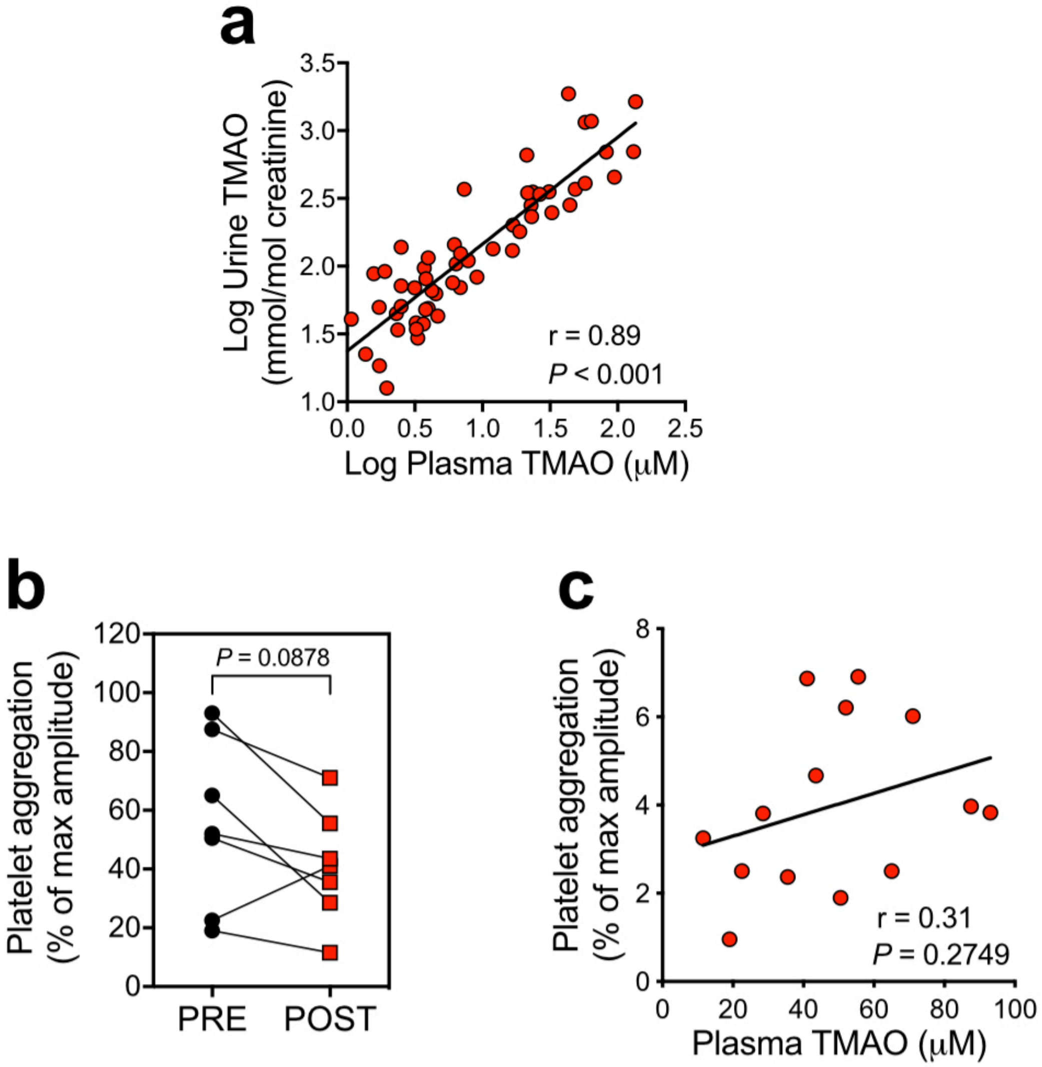

Supplementary Fig. 5 | Effect of raw garlic juice on plasma–urine TMAO correlation and platelet aggregation in TMAO producers (n=7) who received an intervention of garlic juice (55 mL, equivalent to 48 mg of allicin/day) for 1 week, following which they were subject to OCCT. (a) Pearson's correlation of log plasma and urine TMAO, (b) platelet aggregation; and (c) Pearson's correlation of plasma TMAO level and platelet aggregation. Statistical analysis was performed using the two-tailed paired Student's t-test.

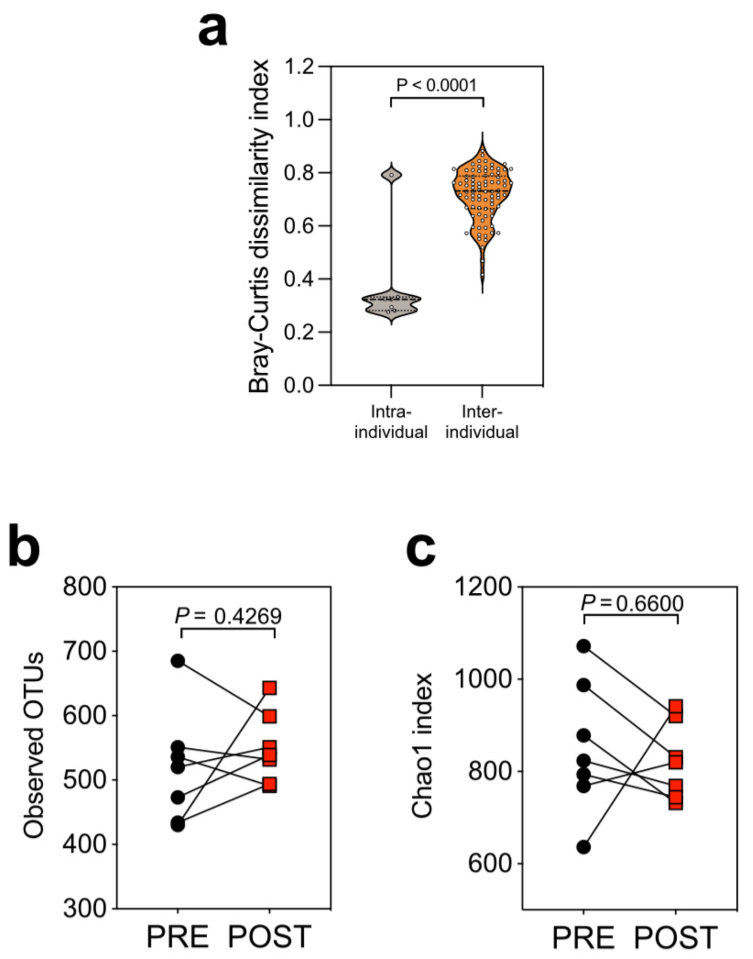

Supplementary Fig. 6 | Effect of raw garlic juice on fecal microbiota in healthy TMAO-producing participants. (a) Bray-Curtis dissimilarity, α-diversity index (b) observed OTUs, and (c) Chao1 index. Statistical analyses were performed using the two-tailed unpaired Student's t-test for dissimilarity index and paired Student's t-test for α-diversity indices.

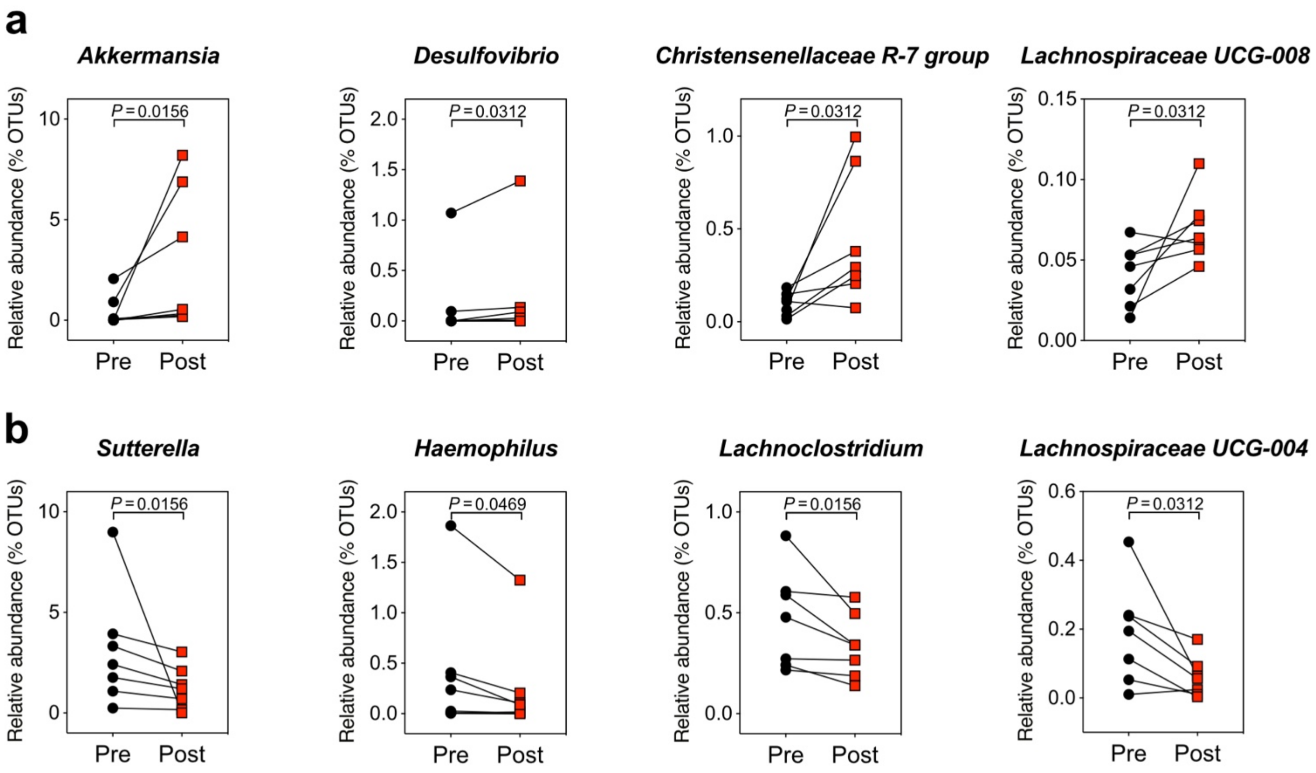

Supplementary Fig. 7 | Effect of raw garlic juice on the fecal microbiota at the genus level in TMAO producers (n=7) who received an intervention of garlic juice (55 mL, equivalent to 48 mg of allicin/day) for 1 week, followed by OCCT. (a) Genera exhibiting an increase in relative abundance, (b) Genera exhibiting a decrease in relative abundance after the intake of raw garlic juice for 1 week. Statistical analysis was performed using the paired-Wilcoxon signed-rank test.

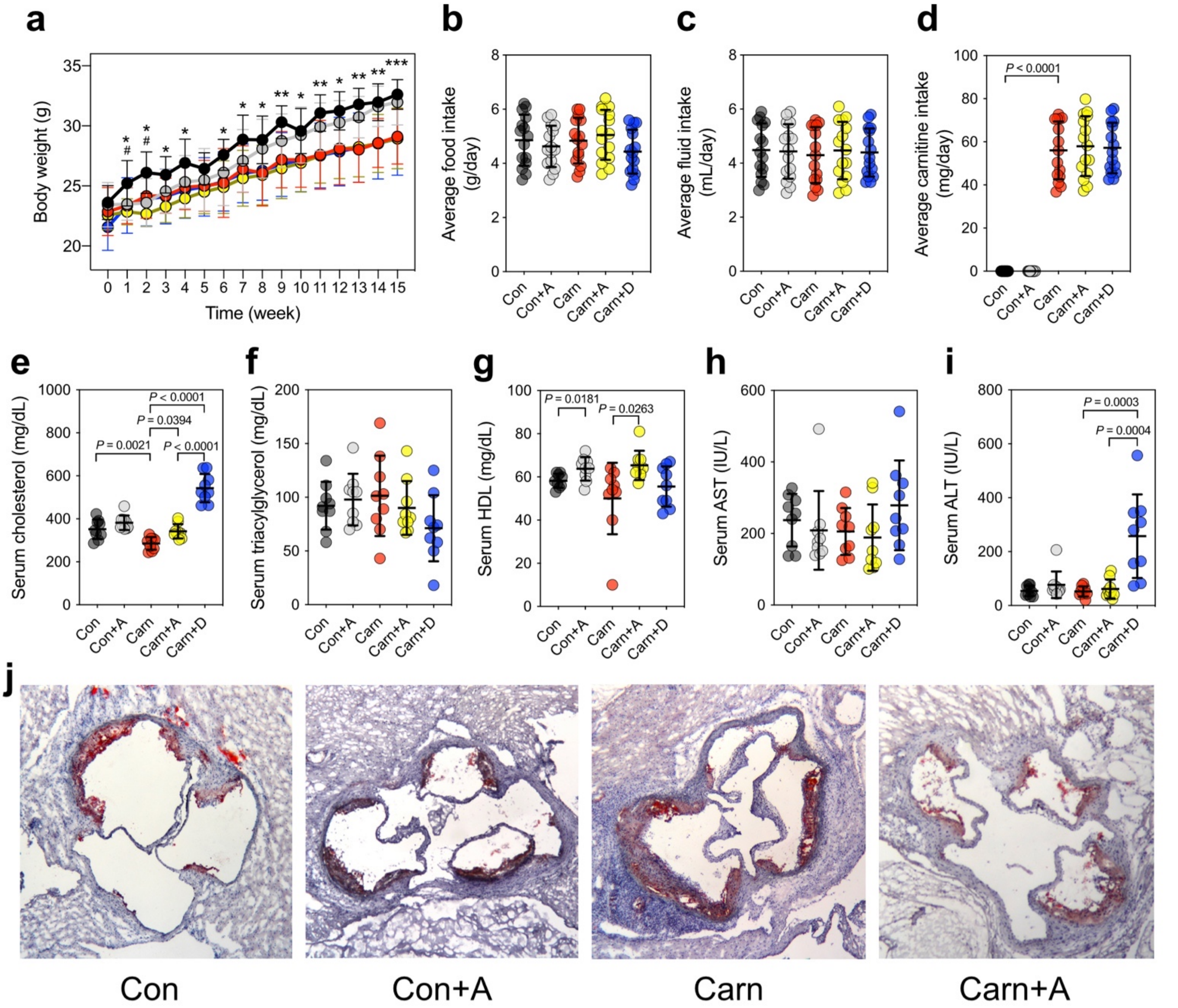

Supplementary Fig. 8 | Effect of allicin and carnitine on ʟ-carnitine-induced atherosclerosis in ApoE$^{-/-}$ mice. (a) Changes in body weight; (b) average food intake; (c) average fluid intake; (d) average carnitine intake; (e) serum cholesterol; (f) triacylglycerol; (g) high-density lipoprotein (HDL); (h) aspartate aminotransferase (AST); (i) alanine aminotransferase (ALT); and (j) representative image of atherosclerotic lesions in the aortic root. Dot plots are expressed as the mean ± SD. Statistical analyses were performed using the unpaired two-tailed Student's t-test Con vs. Con + A group (#, P < 0.05), Con vs. Carn group (*, P < 0.05; **, P < 0.01; and ***, P < 0.001); one-way ANOVA with Tukey's range test for comparisons Carn vs. Carn + A group, Carn vs. Carn + D group, and Carn + A vs. Carn + D group.

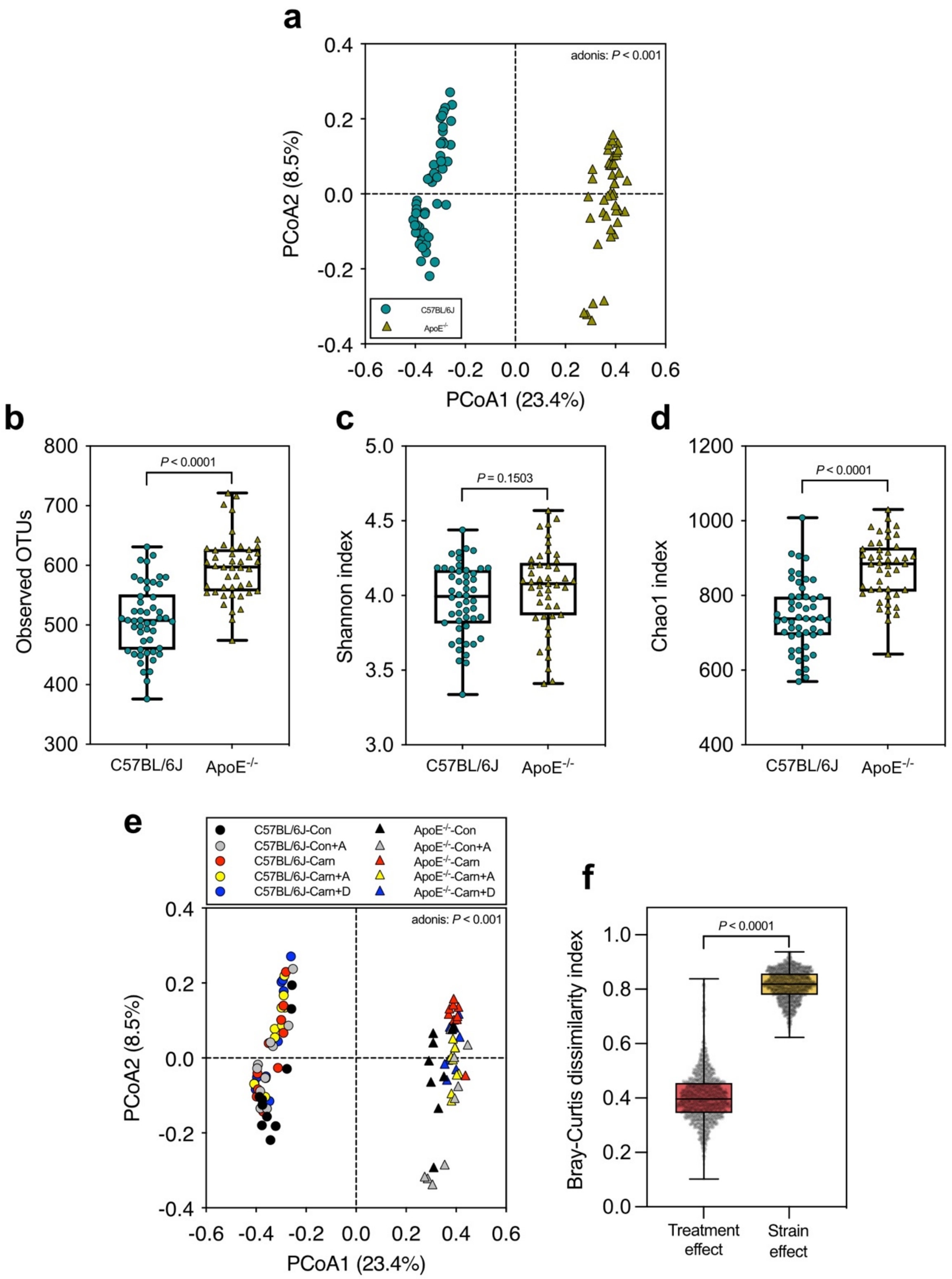

Supplementary Fig. 9 | C57BL/6J and ApoE$^{-/-}$ mice have different gut microbial composition based on α- and β-diversity. The effect of the mouse strain was more prominent on gut microbial composition than the treatment effect. (a) principal coordinate analysis (PCoA) plot with Bray−Curtis dissimilarity comparing C57BL/6J and ApoE$^{-/-}$ mice; α-diversity indices (b) observed OTUs; (c) Shannon index; (d) Chao1 index. (e) PCoA plot with Bray−Curtis dissimilarity comparing C57BL/6J and ApoE$^{-/-}$ mice with different treatment; and (f) Bray−Curtis dissimilarity index of treatment and mouse strain effect. Statistical analysis was performed using the unpaired two-tailed Student's t-test and presented with a *P*-value. Box plots display median, quartiles (boxes), and range (whiskers).

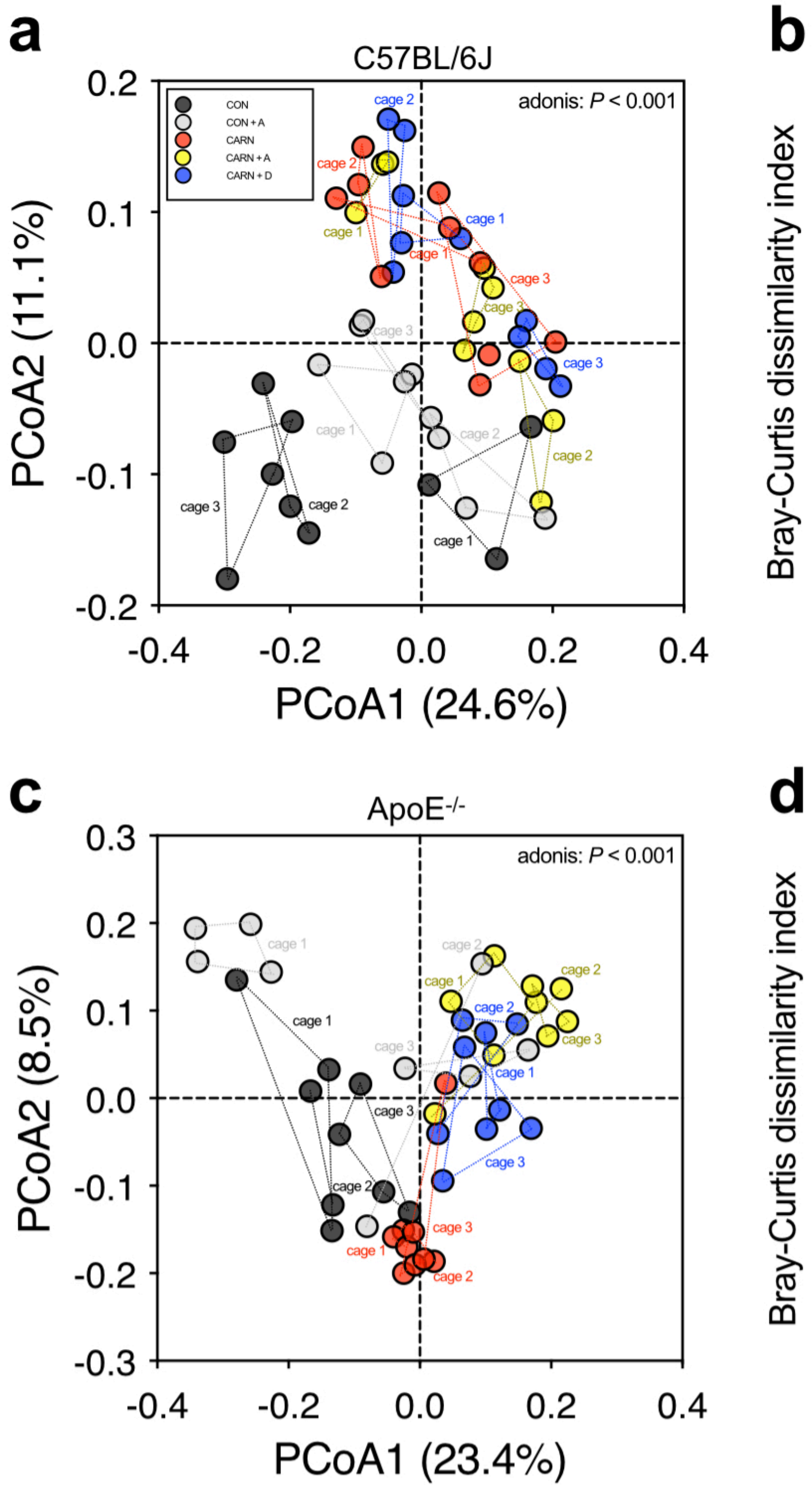

Supplementary Fig. 10| Treatment effects on the gut microbiota were more prominent than the cage effect in both C57BL/6J and ApoE$^{-/-}$ mice. (a) principal coordinate analysis (PCoA) plot with Bray−Curtis dissimilarity of C57BL/6J mice; (b) Bray−Curtis dissimilarity index of C57BL/6J mouse fecal microbiota based on intra-cage, intra-group-inter-cage, and inter group; (c) PCoA plot with Bray−Curtis dissimilarity of ApoE$^{-/-}$ mice; (d) Bray−Curtis dissimilarity index of ApoE$^{-/-}$ mouse fecal microbiota based on intra-cage, intra-group-inter-cage, and inter group. Statistical analysis was performed using the unpaired two-tailed Student's t-test and presented with a *P*-value. Box plots display median, quartiles (boxes), and range (whiskers).

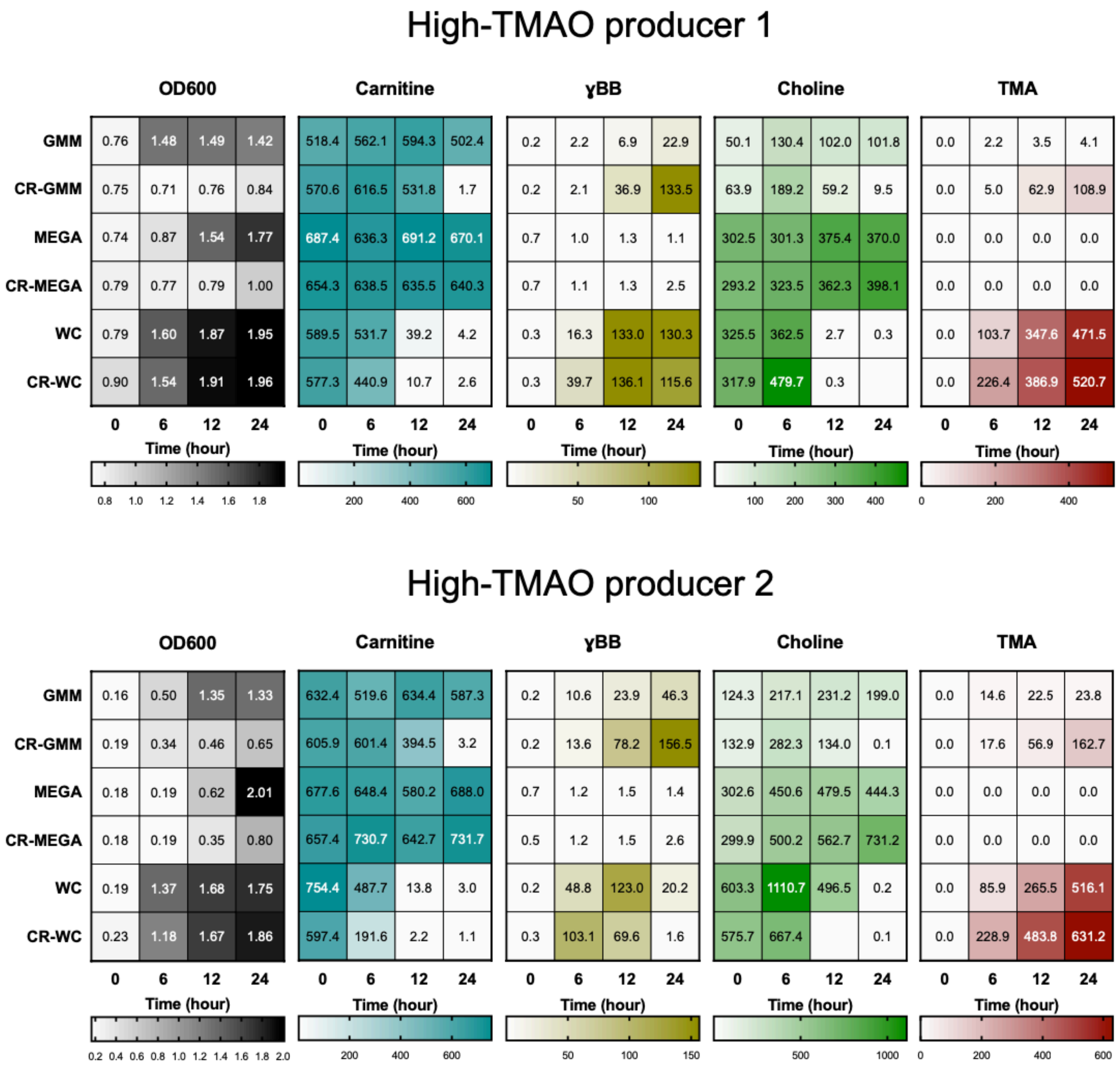

Supplementary Fig. 11 | Comparison of the TMA-producing ability in different media and its carbon-reduced media supplemented with ʟ-carnitine and inoculated with feces from two high-TMAO producers. The Wilkins–Chalgren (WC) medium and its carbon-reduced medium exhibited the highest concentration of TMA after 24 h of culture. GMM: gut microbiota medium; MEGA: mega medium; WC: Wilkins–Chalgren medium; CR-GMM: carbon-reduced gut microbiota medium; CR-MEGA: carbon-reduced MEGA medium; CR-WC: carbon-reduced Wilkins–Chalgren medium.

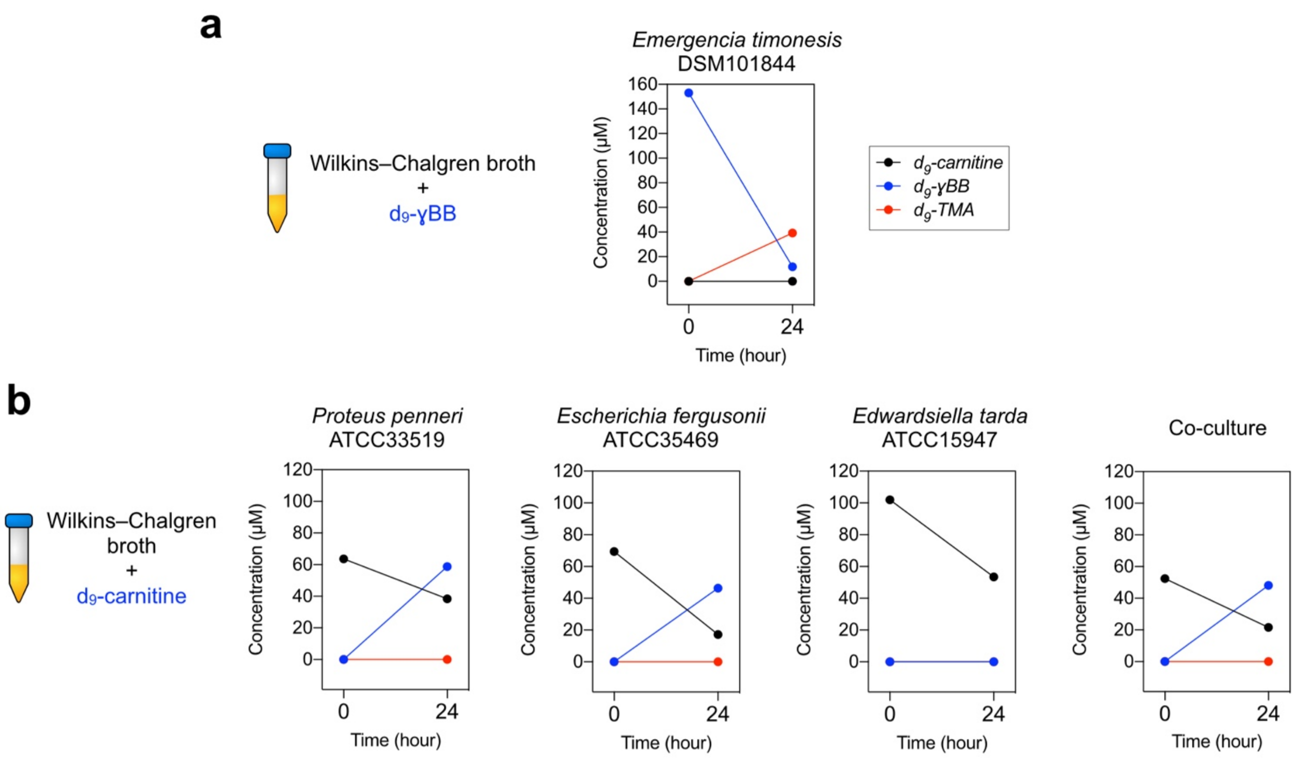

Supplementary Fig. 12 | Utilization of d_9-γBB and d_9-carnitine by single bacterial species and their co-culture. The utilization of (a) d_9-γBB by *Emergencia timonensis* DSM101844 for production of d_9-TMA in Wilkins–Chalgren (WC) broth supplemented with d_9-γBB, and the utilization of (b) d_9-carnitine by *Proteus penneri* ATCC33519, *Escherichia fergusonii* ATCC35469, *Edwardsiella tarda* ATCC15947, and their co-culture in WC supplemented with d_9-carnitine.

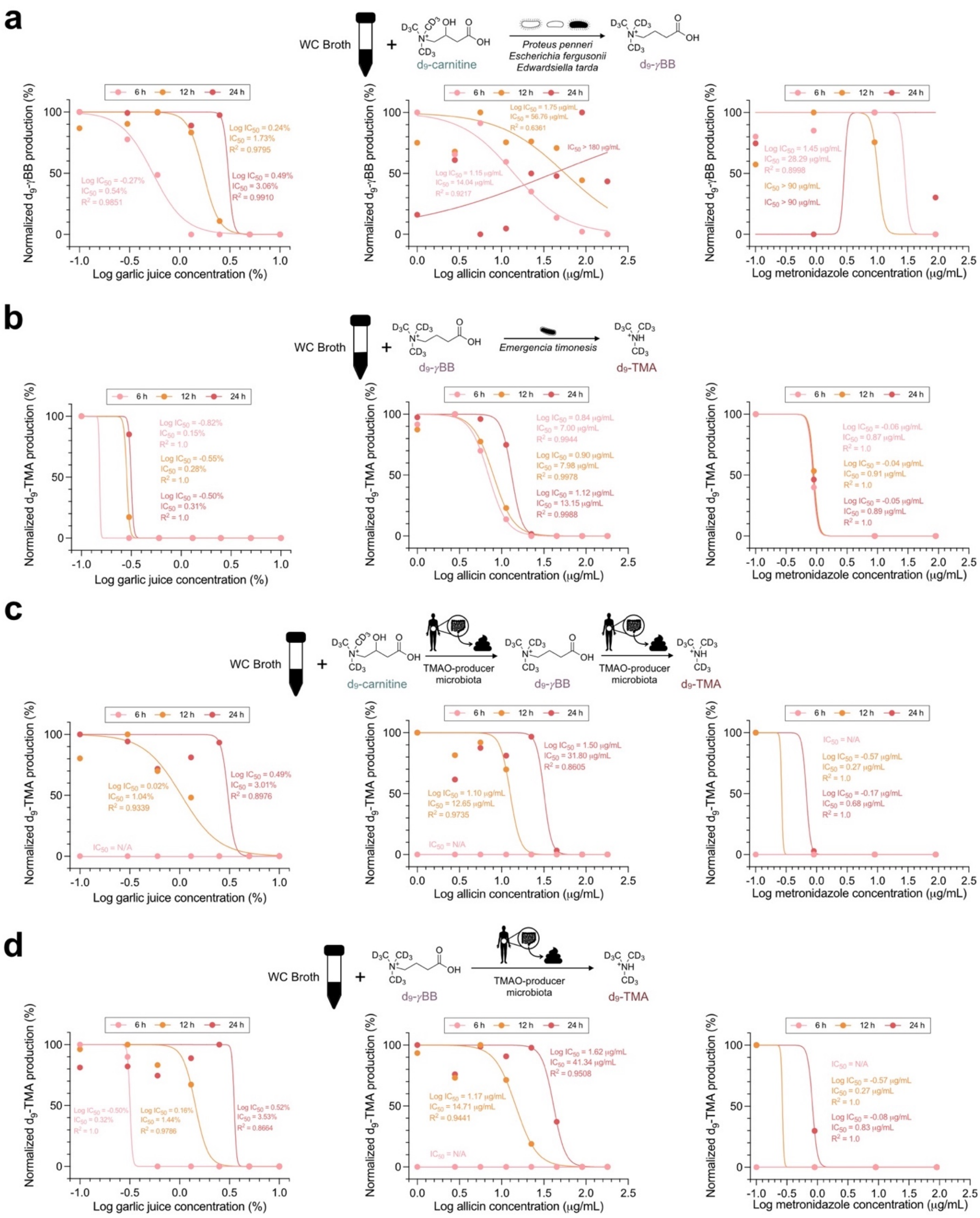

Supplementary Fig. 13 | Half maximal inhibitory concentration (IC_{50}) of garlic juice, allicin, and metronidazole to inhibit the formation of d_9-γBB and d_9-TMA in vitro after inoculation of TMA/γBB-producing bacteria and ex vivo after inoculation of the feces from high-TMAO producers. (a) inhibitory effect of garlic juice, allicin, and metronidazole on carnitine → γBB bacteria (co-culture of *Proteus penneri*, *Escherichia fergusonii*, and *Edwardsiella tarda*) in Wilkins–Chalgren (WC) broth supplemented with d_9-carnitine; (b) inhibitory effect on the bacteria converting γBB to TMA (*Emergencia timonensis*) in WC broth supplemented with d_9-γBB. (c) Inhibitory effect on the high-TMAO-producing gut microbiome in WC broth supplemented with d_9-carnitine and (d) d_9-γBB. Half-maximal inhibitory concentration (IC_{50}) values were determined using nonlinear regression at 6, 12, and 24 h.

Supplementary Table 1. | Effect of raw garlic juice on plasma biochemistry of TMAO producers (n=7) who received garlic juice intervention (55 mL, equivalent to 48 mg of allicin/day) for 1 week.

Blood biochemistry [1]	Garlic Juice Intervention [2]		*P*-value [3]	Normal ranges
	PRE (n = 7)	POST (n = 7)		
Glucose-AC (mg/dL)	78.0 ± 12.4	80.1 ± 6.9	0.5757	65-100 (mg/dL)
AST (U/L)	16.9 ± 3.4	21.1 ± 5.6	0.0105	<40 (U/L)
ALT (U/L)	9.6 ± 8.6	13.4 ± 9.4	0.0012	<41 (U/L)
BUN (mg/dL)	12.9 ± 3.3	11.2 ± 1.0	0.2615	6-20 (mg/dL)
Creatinine (mg/dL)	0.8 ± 0.2	0.8 ± 0.1	0.1723	0.7-1.2 (mg/dL)
T-Cholesterol (mg/dL)	176.6 ± 33.5	189.4 ± 50.0	0.3430	<200 (mg/dL)
Triglyceride (mg/dL)	78.9 ± 20.7	71.6 ± 24.5	0.2846	<150 (mg/dL)
HDL-C (mg/dL)	64.1 ± 16.3	66.4 ± 17.1	0.0703	>40 (mg/dL)
LDL-C (mg/dL)	106.6 ± 29.9	122.1 ± 49.4	0.2511	<100 (mg/dL)
CRP-C (mg/dL)	0.04 ± 0.03	0.05 ± 0.05	0.3559	<0.50 (mg/dL)

[1] Abbreviation: AST, Aspartate aminotransferase; ALT, Alanine aminotransferase; BUN, Blood urea nitrogen; HDL-C, High density lipoprotein cholesterol; LDL-C, Low density lipoprotein cholesterol, CRP-C, C reactive protein.

[2] Blood biochemistry data of healthy subjects are shown above. All data are presented as mean ± SD.

[3] P-value calculated using the two-tailed paired Student's t-test comparing PRE and POST garlic juice intervention are shown.

Supplementary Table 2 | Resources information

Resource or reagent	Source	Identifier
**Bacterial strains**		
Proteus penneri CDC 1808-73	ATCC	33519
Escherichia fergusonii CDC 0568-73	ATCC	35469
Edwardsiella tarda CDC 1483-59	ATCC	15947
Emergencia timonensis SN18	Leibniz Institute	DSM101844
**Chemicals**		
3,3-dimethyl-1-butanol (DMB)	Sigma-Aldrich	Cat#183105
Adenosine 5′-diphosphate sodium salt	Sigma-Aldrich	Cat#A2754
Allicin	Panyod et al., 2020	N/A
AMPure XP beads	Beckman Coulter	A63881
Carboxymethyl cellulose	Sigma-Aldrich	Cat#C4888
DL-Carnitine-(trimethyl-d_9) hydrochloride	Sigma-Aldrich	Cat#729868
Gelatin peptone	Sigma-Aldrich	Cat#70176
Glucose	Bioshop	Glu501.1
Hemin	Sigma-Aldrich	Cat#H9039
KAPA HiFi HotStart ReadyMix	Roche	KK2602
L-Carnitine-(methyl-d_3) hydrochloride	Sigma-Aldrich	Cat#616737
L-Arginine	Sigma-Aldrich	Cat#AS006
L-carnitine	General Nutrition Centers	802420
L-carnitine hydrochloride	Sigma-Aldrich	Cat#C0283
L-cysteine	Sigma-Aldrich	Cat#168149
Menadione	Sigma-Aldrich	Cat#M5625
Nextera XT Index Kit	Illumina	FC-131-1096
Oil red O	Sigma-Aldrich	Cat#O0625
Propylene glycol (1,2-Propanediol)	Sigma-Aldrich	Cat#134368
Sodium chloride	Bioshop	SOD002.1
Sodium citrate	Mallinkrodt	0754-12
Sodium pyruvate	Sigma-Aldrich	Cat#P2256

Trimethyl-$^{13}C_3$-amine hydrochloride	Sigma-Aldrich	Cat#591599
Trimethyl-d_9-amine hydrochloride	Sigma-Aldrich	Cat#613843
Trimethylamine-$^{13}C_3$ N-Oxide Hydrate	Toronto Research Chemicals	T795793
Trimethylamine-d_9 N-Oxide	Sigma-Aldrich	Cat#791628
Tryptone	Sigma-Aldrich	Cat#70169
Yeast extract	Bioshop	YEX401.500
γ-Butyrobetaine-d_9 hydrochloride	Medical Isotopes	D39211
**Commercial assays**		
Spotchem II reagent strips: Total cholesterol	Arkray Inc.	77162
Spotchem II reagent strips: Triglyceride	Arkray Inc.	77163
Spotchem II reagent strips: High-density lipoprotein (HDL-c)	Arkray Inc.	77181
Spotchem II reagent strips: Aspartate transaminase (AST)	Arkray Inc.	77169
Spotchem II reagent strips: Alanine aminotransferase (ALT)	Arkray Inc.	77170
QIAamp PowerFecal DNA Kit	QIAGEN	12830-50
**Deposited data**		
16S rRNA gene sequencing of human and mouse fecal microbiota	This study	NCBI BioProject: PRJNA661156 and SRA: SRS7316558, SRS7317051, and SRS7315455
**Mouse strains**		
Mouse: C57BL/6JNarl	National Laboratory Animal Center, Taiwan	RMRC11005
Mouse: $ApoE^{-/-}$ ($Apoe^{tm1Unc}$)	Original from The Jackson Laboratory	002052
**Oligonucleotides**		
Forward: 5′-TCG TCG GCA GCG TCA GAT GTG TAT AAG AGA CAG CCT ACG GGN GGC WGC AG-3′	Illumina, 2014	N/A

Reverse: 5′-GTC TCG TGG GCT CGG AGA TGT GTA TAA GAG ACA GGA CTA CHV GGG TAT CTA ATC C-3′	Illumina, 2014	N/A
**Software**		
Image J software (Version 1.8.0)	Schneider et al., 2012	https://imagej.nih.gov/ij/
16S Bacteria/Archaea SOP v1 of the Microbiome Helper workflows	Comeau et al., 2017	https://github.com/mlangill/microbiome_helper
VSEARCH v2.1.2.	Rognes et al., 2016	https://github.com/torognes/vsearch
QIIME v.1.9.1.	Caporaso et al., 2010	http://qiime.org/
Graphpad Prism (version 9.2.0)	GraphPad Software Inc	http://www.graphpad.com
R (version 3.6.1) with vegan and heatmap3 packages	R Foundation	https://www.r-project.org<br>https://cran.r-project.org/web/packages/vegan/index.html<br>https://cran.r-project.org/web/packages/heatmap3/heatmap3.pdf
R Studio (version 1.2.5001)	R Studio	https://www.rstudio.com

Supplementary Table 3 | Gut microbiota medium (GMM) and its carbon-reduced medium recipe

Gut microbiota medium (GMM)	Regular medium (1 L)	Carbon-reduced medium (1 L)
Tryptone Peptone	2 g	2 g
Yeast Extract	1 g	1 g
D-glucose	0.4 g	-
L-cysteine	0.5 g	0.5 g
Cellobiose	1 g	-
Maltose	1 g	-
Fructose	1 g	-
Meat Extract	5 g	5 g
KH_2PO4	100 mL	100 mL
$MgSO_4$-$7H_20$	0.002 g	0.002 g
$NaHCO_3$	0.4 g	0.4 g
$NaCl_2$	0.08 g	0.08 g
$CaCl_2$	1 mL	1 mL
Vitamin K (menadione)	1 mL	1 mL
$FeSO_4$	1 mL	1 mL
Histidine Hematin Solution	1 mL	1 mL
Tween 80	2 mL	2 mL
ATCC Vitamin Mix	10 mL	10 mL
ATCC Trace Mineral Mix	10 mL	10 mL
Acetic acid	1.7 mL	1.7 mL
Isovaleric acid	0.1 mL	0.1 mL
Propionic acid	2 mL	2 mL
Butyric acid	2 mL	2 mL
Resazurin	4 mL	4 mL

Supplementary Table 4 | MEGA medium (MEGA) and its carbon-reduced medium recipe

MEGA medium (MEGA)	Regular medium (500 mL)	Carbon-reduced medium (500 mL)
Tryptone Peptone	5 g	5 g
Yeast Extract	2.5 g	2.5 g
D-(+)-Glucose	1 g	-
L-Cysteine HCl	0.25 g	0.25 g
1 M Potassium Phosphate Buffer, pH 7.2	50 mL	50 mL
TYG Salt Solution (0.5 g of $MgSO_4{\cdot}7H_2O$; 10.0 g of $NaHCO_3$; 2 g of NaCl in 1L of H_2O)	20 mL	20 mL
Vitamin K Solution	500 µL of 1 mg/mL	500 µL of 1 mg/mL
0.8 % $CaCl_2$	500 µL	500 µL
$FeSO_4{\cdot}7H_2O$	500 µL of 0.4 mg/mL	500 µL of 0.4 mg/mL
Resazurin	2 mL of 0.25 mg/mL	2 mL of 0.25 mg/mL
Histidine-Hematin	500 µL	500 µL
dH_2O	150 mL	150 mL
D-(+)-Cellobiose	0.5 g	0.5 g
D-(+)-Maltose	0.5 g	0.5 g
D-(-)-Fructose	0.5 g	0.5 g
Soluble Starch	12.5 mL of 2%	12.5 mL of 2%
Tween 80	1 mL of 25%	1 mL of 25%
Meat Extract	2.5 g	2.5 g
Trace Mineral Supplement (ATCC)	5 mL	5 mL
Vitamin Supplement (ATCC)	5 mL	5 mL
SCFA Supplement Stock (Modified Recipe) (Final Moles/L in Media: Acetic acid (0.0297); Isovaleric acid(0.0009); Propionic acid (0.0081); and N-Butyric acid (0.0044))	3.75 mL	3.75 mL

Supplementary Table 5 | Wilkins–Chalgren medium (WC) and its carbon-reduced medium recipe

Wilkins-Chalgren medium (WC)	Regular medium (1 L)	Carbon-reduced medium (1 L)
Tryptone	10 g	10 g
Gelatin peptone	10 g	10 g
Yeast extract	5 g	5 g
Glucose	1 g	-
Sodium chloride	5 g	5 g
L-Arginine	1 g	1 g
Sodium pyruvate	1 g	1 g
Menadione	0.0005 g	0.0005 g
Hemin	0.005 g	0.005 g
